# Drastic Variations in Chemical Composition of Organic Inputs: Implications for Organic Fertilization

**DOI:** 10.1021/acs.est.5c06493

**Published:** 2025-07-28

**Authors:** Jill Bachelder, Matthias Wiggenhauser, Lenny H. E. Winkel, Emmanuel Frossard, Julie Tolu

**Affiliations:** † 27219 ETH Zurich, Swiss Federal Institute of Technology, Department of Environment Systems Sciences (D-USYS), Institute of Biogeochemistry and Pollutant Dynamics (IBP), Group of Inorganic Environmental Geochemistry, Universitätstrasse 16, 8092 Zurich, Switzerland; ‡ 28499 Eawag, Swiss Federal Institute of Aquatic Science and Technology, Department of Water Resources and Drinking Water (W+T), Überlandstrasse 133, 8600 Dübendorf, Switzerland; § ETH Zurich, Swiss Federal Institute of Technology, Department of Environment Systems Sciences (D-USYS), Institute of Agricultural Sciences (IAS), Group of Plant Nutrition, Eschikon 33, 8315 Lindau, Switzerland

**Keywords:** organic amendments, fertilization, trace elements, micronutrient, contamination, size exclusion chromatography, ICP-MS/MS, pyrolysis-GC/MS

## Abstract

Soil amendment with organic inputs is gaining importance with the ongoing shift toward circular economies. While these inputs can fertilize soils with micronutrients such as zinc (Zn), it is crucial to prevent potential contamination stemming from Zn accumulation in soils or crop uptake of toxic elements such as cadmium (Cd). While both organic matter (OM) composition and Zn and Cd speciation are key factors controlling Zn and Cd fate in soil-plant systems, these factors remain largely uncharacterized in many commonly used organic inputs. We unveil substantial differences in water-soluble Zn and Cd speciation and the OM molecular composition in various organic inputs. We found that plant-based organic inputs (e.g., green manures, lignified crop residues) were characterized by lower Zn and Cd concentrations and by enrichment in rapidly degradable OM. In contrast, animal-waste-based inputs (e.g., farmyard manures, composts) were characterized by relatively higher Zn and Cd concentrations and more degraded and resistant OM. Combining size exclusion chromatography coupled to atomic mass spectrometry with geochemical equilibrium modeling (WHAM VII) showed that water-soluble Zn in plant-based inputs was mostly bound to lower-molecular-weight OM while Cd was associated with higher-molecular-weight OM. This suggests potentially higher plant availability of water-soluble Zn compared to Cd. In contrast to plant-based inputs, the majority of animal-waste-based inputs showed water-soluble Zn and Cd primarily in the inorganic aqueous form (e.g., Zn^2+^, ZnOH^+^, Cd^2+^, CdOH^+^) and/or bound to higher-molecular-weight OM. The in-depth characterization of organic inputs provided here establishes a foundation for better understanding of the fate of Zn and Cd in circular agroecosystems.

## Graphical abstract

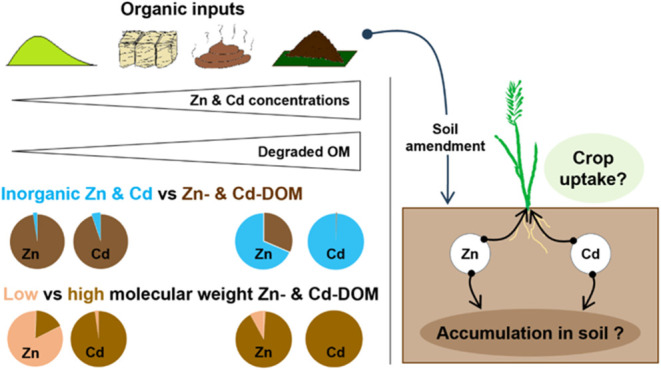

## Introduction

Agricultural systems must evolve to meet the demands of growing populations while minimizing impacts on the environment.^1,2^ A promising approach involves transitioning toward circular systems that utilize organic inputs to recycle nutrients back into soils, reducing reliance on synthetic mineral fertilizers.^3,4^ This strategy is expected to reduce land use and greenhouse gas emissions, while still supporting food production for expanding populations.^3^ Organic inputs offer several agronomic benefits. They enhance soil aeration, water and nutrient (e.g., nitrogen) retention,^5–7^ and increase the concentration of essential micronutrients in soil such as zinc (Zn).^8–11^ However, their application also poses risks.^12^ Organic inputs can lead to metal (e.g., Zn) accumulation, potentially disrupting soil functions if concentrations exceed critical thresholds.^13–15^ They may also introduce highly toxic, nonessential elements such as cadmium (Cd), along with other pollutants, which can accumulate in crops and soils.^12,16–18^

To effectively replace synthetic fertilizers, the advantages and risks of organic input application must be better understood, including the balance between the addition of essential micronutrients (e.g., Zn) and harmful pollutants (e.g., Cd). This requires a detailed biogeochemical characterization of the diverse organic inputs commonly available in agriculture.^19,20^ Element speciation and the composition of organic matter (OM) are key factors influencing the (im)­mobilization and plant uptake of elements like Zn and Cd in soils.^15,21–23^ Thus, understanding how these parameters vary across different organic inputs is critical for predicting their impact on Zn and Cd behavior in soil-plant systems.

Using synchrotron X-ray absorption spectroscopy, previous studies have shown that Zn in organic wastes (e.g., sewage sludge, poultry manure, pig slurry, and their composts) exists in several forms: bound to OM (through O-, and phosphate-containing functional groups), iron (Fe) or phosphate (PO_4_^3–^) minerals, and as nanostructured zinc sulfides (ZnS).^19,20,24^ In plants, Zn is primarily bound to PO_4_^3–^ and to OM via O- and N-containing functional groups.^25–28^ Geochemical multisurface modeling further revealed that >85% of water-soluble Zn in composts exists as Zn complexes with dissolved organic matter (DOM). The remainder is present as free Zn^2+^ and inorganic complexes (e.g., ZnOH^+^, ZnSO_4_), which are more mobile and plant-available,^21,22^ although low-molecular-weight Zn-DOM complexes can also be mobile and taken up by plants.^29^ While these studies provide important insights into Zn speciation and its stability in select organic inputs (typically 1–3 input types per study), comprehensive comparisons across a broader spectrum of organic inputs—such as various green manures, farmyard manures, slurries, and composts— are still lacking. For Cd, solid-phase speciation studies have been limited to hyperaccumulating plants, mature rice straw, and cacao branches,^30–35^ and suggest binding of Cd to OM through O, N, or reduced S functional groups in green manure and lignified residues. Otherwise, no data exist for Cd speciation in the organic inputs. This is because high detection limits hinder the application of synchrotron techniques for Zn and Cd analysis in organic inputs. Yet, it is essential to investigate how Zn and Cd speciation vary across the organic inputs available for use in agricultural systems that shift toward circularity, to better guide the evaluation of their associated advantages and potential risks, using, e.g., pot or field experiments.

Beyond introducing toxic or essential elements to soils, organic input application can influence the behavior of soil-bound elements through addition of OM.^8,10,36^ Studies have suggested that soluble organic compounds, released upon organic input application and decomposition in soils, promote soil Zn and Cd solubilization and uptake by plants.^8,10^ Indeed, these compounds can act as organic ligands that complex Zn and Cd, thus mobilizing species previously immobilized in the soil solid phases.^21,22^ However, the type and quantity of ligands released vary with OM composition and degradability.^37^ While some studies have characterized OM composition in specific organic inputs to assess their persistence in soils,^38–40^ comprehensive comparisons across diverse organic inputs remain rare.

This study aimed to evaluate the extent to which different organic inputs exhibit distinct chemical characteristics that are relevant to the availability of Zn and Cd to plants and their accumulation in soils. To this end, we characterized OM molecular composition and water-soluble Zn and Cd speciation, alongside conventional bulk parameters (i.e., pH and elemental concentrations), in a wide variety of organic inputs used in agricultural systems (*n* = 28). OM molecular composition was determined using pyrolysis-gas chromatography/mass spectrometry (Py-GC/MS), which provides semiquantitative data on pyrolytic products of OM that are specific to different biochemical OM classes (e.g., carbohydrates, proteins, lipids) and are indicative of OM degradability.^41^ For Zn and Cd speciation, our analyses focused on the water-soluble pool, representing the portion most likely to enter the soil solution postapplication and to be potentially more available for plants.^21,23^ This important fraction can account for up to 50% of total Zn or Cd in organic inputs.^42–47^ Two approaches were used to evaluate functional Zn and Cd speciation, i.e., to characterize Zn and Cd forms that are environmentally relevant.^48^ Geochemical equilibrium modeling (WHAM VII)^49^ was used to estimate the proportions of Zn and Cd present as inorganic species (i.e., Zn^2+^, Cd^2+^, and aqueous inorganic complexes such as complexes with −OH^-^ or −SO_4^2-^_), known to be mobile and plant-available,^21,22^ versus the proportions of Zn and Cd bound to DOM. Size exclusion chromatography (SEC) combined with online detections of OM (by ultraviolet —UV—) and multiple elements (by inductively coupled plasma tandem mass spectrometry —ICP-MS/MS—)^50^ was used to estimate the proportions of Zn and Cd associated with different size and chemical fractions of DOM. SEC was used because previous studies have shown that Zn and Cd complexes with low-molecular-weight organic compounds are mobile and can be taken up by plants,^22,29,51–53^ and SEC-ICP-MS has previously been successful in estimating trace element binding to low- versus high-molecular-weight OM in soils, plants, composts, and groundwater samples.^54–57^ Based on our comprehensive chemical profiling of different organic inputs, we discuss potential increases in the plant-available pool versus accumulation of Zn and Cd in soils postapplication.

## Experimental Section

### Sampling and Pretreatments

Twenty-eight organic inputs were collected from six sites in Switzerland. Sample identification, sampling location, sampling procedure, and storage time are detailed in Table S1, Notes S1, and S2. Prior to analysis, all samples were freeze-dried and finely ground using a Quiagen TissueLyzer ball mill with tungsten-carbide beakers. Eleven inputs were processed in replicates of three or more to evaluate homogeneity (Table S1).

### Characterization of OM Molecular Composition

OM composition of organic inputs was characterized by Py-GC/MS (Method S1 and Table S2).^41^ 266 pyrolytic (Py-) products of OM from different biochemical classes were detected (Table S3). For each Py-product, the relative abundance was calculated by taking the ratio between its peak area (signal intensity) and the total peak area of all Py-products. The peak area of each identified organic compound is proportional to the concentration of the organic compounds from which the pyrolytic compounds were derived, with the sum of identified peak areas correlating positively with total C concentrations (Figure S1). To constrain the data presentation and associated discussion, and to avoid overinterpretation of individual compounds, the 266 identified Py-products were reduced to 17 groups based on their similarities in molecular structure and/or the OM biochemical class from which they were derived. The freshness index of carbohydrates was calculated as fresh carbohydrates (levosugars, anhydrosugars) divided by the sum of degraded carbohydrates (cyclohexanone, cyclopentanone, furans, pyrans). The freshness index of N compounds was calculated as fresh N (proteins) divided by the sum of degraded N compounds (aromatic N compounds, alkylamides, alkanenitriles). Reproducibility, which was measured as relative standard deviation (RSD) of the identified peaks in samples analyzed in triplicate, was satisfactory. The RSD was on average 9 ± 11%, with 90 and 95% of the RSD values being within 18 and 23%, respectively. The high RSD values stem from the low signal of terpenoids in green manure samples.

### Total Elements Quantification

Total carbon (C), nitrogen (N), and sulfur (S) concentrations were measured with an elemental analyzer (Vario PYRO cube, Elementar GmbH). Zn, Cd, and other elements were quantified after digestion in 8 M HNO_3_ using a high-pressure single-reaction microwave (turboWave, MWS GmbH, Heerbrugg; Method S2). Element quantification in the digests was performed using an Agilent 7500ce ICP-MS or 8900 ICP-MS/MS instrument (Method S3). Quality control was performed by digesting and analyzing the solid certified reference material (CRM) WEPAL 232 (eucalyptus leaves) and analyzing the liquid CRM NIST 1643f (freshwater). Recoveries between 80–120% and 90–120% were obtained for the WEPAL 232 and NIST 1643f, respectively (Tables S4 and S5).

### Water Extraction and Determination of Extract Composition and Zn and Cd Speciation

#### Extraction, Subsampling, and pH

The water-soluble pool of the organic inputs was extracted using ultrapure water (>18.2 MΩ cm, Nanopure DiamondTM system) at different solid:liquid (S:L) ratios depending on organic inputs (Table S6). Increased S:L were used for samples whose SEC-UV-ICP-MS/MS signals (Zn and Cd speciation analysis) were too low at original S:L of 1:100. The organic input and water mixtures were shaken (overhead shaker; 2 h; room temperature) and centrifuged (2700*g*; 20 min). The water extracts were filtered (0.45 μm; Nylon membrane), and a 200 μL subsample was immediately analyzed for pH (Metrohm, Biotrode). Four additional subsamples were taken: (1) ∼3 mL was diluted 10-fold with ultrapure water and frozen for quantification of dissolved organic carbon (DOC) within 2 weeks; (2) 4 mL was frozen for major ions quantification within 2 weeks; (3) 1–3 mL was acid-digested within 1 week and kept at 4 °C for element quantification; and (4) 1 mL was analyzed within 1–2 days for Zn and Cd speciation by SEC-UV-ICP-MS/MS.

#### DOC, DOM, Major Ions, and Elements Quantification

DOC was quantified after acidification with HCl using a Shimadzu TOC-L CSH analyzer, and dissolved organic matter (DOM) was calculated by multiplying the DOC concentrations by a factor of 2.^58,59^ Major ions were measured with a Metrohm 930 Compact IC Flex with chemical suppression except PO_4_^3–^ and NH_4_^+^, which were quantified using the malachite green method and Berthelot’s reaction (Agilent Cary 60 Spectrophotometer), respectively.^60,61^ Organic inputs containing carbonates were identified by applying droplets of 1 M HNO_3_ and observing bubble formation. Samples containing carbonates were analyzed for alkalinity via titration with Titriplex III (0.1 mol L^–1^) by using a Metrohm 809 Titrando ion selective electrode. For element quantification, the water extracts were digested by microwave-assisted acid digestion (Method S2), and the digests were measured by ICP-MS/MS (Method S3, Tables S4 and S5).

#### Zn and Cd Speciation

Zn and Cd speciation in water extracts was measured using size exclusion chromatography (SEC) coupled to both UV and ICP-MS/MS (Method S4).^50^ The intensity chromatograms obtained for UV, C, P, S, Fe, Zn, and Cd were deconvoluted using the peak analyzer function of Origin2021 software.^54^ The *r* ^2^ values of the peak-fit were ≥0.98 for all deconvoluted chromatograms, except for C (*r* ^2^ > 0.5) and Cd (*r* ^2^ > 0.4), due to low peak intensities.

As SEC-UV-ICP-MS/MS does not allow characterization of inorganic aqueous species (e.g., free Zn^2+^ and Cd^2+^), Zn and Cd speciation was predicted using the Windermere Humic Aqueous Model (WHAM VII).^49^ Input parameters included pH, major ion concentrations (Na^+^, Mg^2+^, K^+^, Ca^2+^, NH_4_^+^, F^–^, Cl^–^, NO_3_^–^, PO_4_^3–^, and SO_4_^2-^) and total element concentrations (i.e., Mn, Fe, Ni, Cu, Zn, and Cd) in extracts as well as reactive dissolved organic matter (reactive DOM, called “colloidal fulvic acid” in the model). For samples containing carbonates, alkalinity was added as an input parameter. For all other samples, the partial pressure of carbon dioxide was set to 0.004 atm following studies on soils.^62^ For the input parameter “reactive DOM”, Zn and Cd speciation was modeled considering that 10, 50, 65, and 90% of measured DOM comprised reactive DOM.^63,64^ For the fifth scenario, hereafter referred to as “SEC%”, reactive DOM was calculated for each analyzed organic input based on the SEC-UV-ICP-MS/MS data set, and Zn and Cd speciation was modeled using these estimated reactive DOM proportions as further explained in the Results and Discussion Section. The model output parameters considered were concentrations of Zn^2+^, Cd^2+^, inorganic aqueous Zn and Cd complexes (i.e, ZnOH^+^, Zn­(OH)_2_, ZnSO_4_, ZnCO_3_, ZnCl^+^, ZnHCO_3_^+^, CdOH^+^, Cd­(OH)_2_, CdSO_4_, CdCl^+^, CdCl_2_, CdHCO_3_^+^, CdCO_3_, and Cd­(CO_3_)_2_^2–^), and Zn and Cd bound to reactive DOM.

### Statistical Analyses

Difference in OM molecular composition between organic inputs was explored with principal component analysis (PCA) after converting the OM data set to *z*-scores. Principal components (PCs) with eigenvalues >1 were extracted using Varimax matrix rotation. Factors were considered to drive a PC if their loadings were >0.39.^65^ Differences in bulk parameters, OM composition, and Zn and Cd speciation were also explored using hierarchical cluster analysis (Ward’s linkages based on squared Euclidean distances). Bivariate correlation coefficients were determined as 2-tailed Spearman coefficients. All statistical analyses were done using SPSS Statistics 23 (IBM) and OriginPro 2021 (OriginLab).

## Results and Discussion

### Bulk Properties of Investigated Organic Inputs

For the 28 sampled organic inputs, we first determined bulk properties that are conventionally used to characterize organic inputs (e.g., pH, total C, N, S),^19,20,66^ and evaluated their variability across samples using hierarchical cluster analysis (Table 1). Cluster 1 included all green manure samples and one lignified crop residues and litter (noted "LCR/Litter"), one compost, and one ruminant farmyard manure (FYM). Cluster 2 contained only lignified crop residues and litter , and cluster 4 contained only composts. Cluster 3 represented animal-waste-derived organic inputs and contained all monogastric farmyard manure and slurry samples (noted “monogastric FYM”), all ruminant and ruminant-like farmyard manure samples (noted “ruminant FYM”), and all cattle slurry samples. Note that when selecting five (instead of four) clusters, three monogastric FYM samples grouped into the new cluster, and when selecting six clusters, only one green manure sample formed the new cluster (Table S7).

**Table 1:**
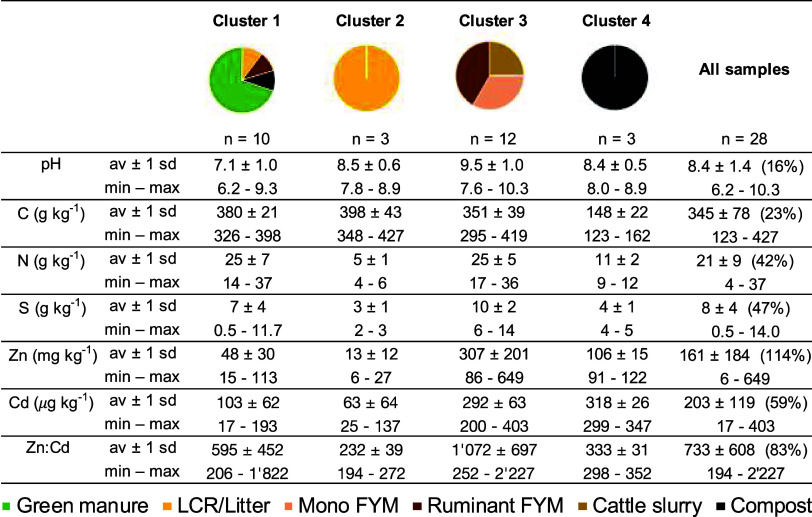
pH and Concentrations of Total Carbon (C), Nitrogen (N), Sulfur (S), Zinc (Zn), and Cadmium (Cd) in the Investigated Organic Inputs, Organized According to the Four Clusters Resulting from Hierarchical Cluster Analysis Performed with the Bulk Properties (i.e., pH and C, N, S, Zn, and Cd Concentrations)^a^

a Zn-to-Cd (Zn:Cd) ratios (not included in the cluster analysis) were calculated by mass. For each parameter, the average and standard deviation (av ± 1 sd) as well as the minimal and maximal (min–max) values of each cluster and of the entire sample set are provided. For the entire sample set, the overall relative standard deviation (RSD, in percent) is provided in parentheses after the average and standard deviation values. The RSD was calculated in percent using the following formula: 100 * standard deviation across all samples, divided by average across all samples. The organic input type “lignified crop residues and litter” is abbreviated as “LCR/Litter,” “monogastric” is abbreviated “mono”, and “farmyard manure” is abbreviated “FYM”.

Cluster 1 (mostly composed of green manure samples) had the lowest pH (7.1 ± 1.0) while cluster 3 (animal-waste-derived organic inputs) had the highest pH (9.5 ± 1.0), aligning with previous studies.^19,67–71^ Total C concentrations spanned 123–427 g (kg)^−1^, with 2–3 times lower average values for cluster 4 (composts) than the other clusters, likely due to OM degradation during the aerobic composting process.^72^ Total N concentrations were 4–37 g kg^–1^, with average values 2–5 times lower in LCR/Litter (cluster 2) and in composts (cluster 4) than other sample clusters. The concentrations of Zn and Cd were 6–649 mg kg^–1^ and 17–403 μg kg^–1^, respectively, which matched the ranges found in the literature (Table S8). Concentrations of both Zn and Cd were on average 2–20 times higher in animal-waste-derived organic inputs (cluster 3) and composts (cluster 4) than in plant-based organic inputs (clusters 1–2). The high Zn concentrations found in animal-waste-derived organic inputs (cluster 3; 86–649 mg kg^–1^) are likely due to mineral Zn present in animal feed as a nutrient.^73,74^ Similar to Zn, lowest Cd concentrations were found in green manures and LCR/Litter, but, in contrast to Zn, animal-waste-derived organic inputs and composts had Cd concentrations similar to one another. Overall, Zn:Cd was 2–5 times larger in animal-waste-derived organic inputs (cluster 3) compared to the other input types. Although this data provides insight into potentially important co-input of Zn and Cd, especially with animal waste (farmyard manure and slurry), a generalization of the trends observed is limited. In green manures and LCR/Litter, Zn and Cd concentrations depend on their concentrations in the soils on which the plants were originally grown and on plant species.^75^ In animal waste, Zn and Cd concentrations depend on the concentrations in the original animal feed, while in composts, they depend on inputs from which the compost was produced.

### Py-GC/MS Analysis Shows Five Main OM Composition Types

Differences in solid-phase OM molecular composition across investigated organic inputs were explored by applying principal component analysis (PCA) to the Py-GC/MS data set (Figure 1 and Tables S9 and S10). The PC1 positive side was driven by aromatic N, phenolic, and (poly)­aromatic compounds, i.e., well-known pyrolytic products of degraded OM,^76,77^ and by *n-*alkanes, *n-*alkenes, alkylamides, and alkanenitriles, i.e., pyrolytic products of cell wall and wax lipids and/or biomacromolecules generally recalcitrant to degradation (Figure 1a).^65,76–84^ On PC 1′s positive side, we also found steroids, terpenoids, and tocopherols, i.e., biomacromolecules that may be degraded by aerobic but not anaerobic microbial activity.^85–87^ PC1′s positive side thus represented degraded and anaerobically resistant OM. On PC2, positive loadings were found for levosugars and anhydrosugars, i.e., specific pyrolytic products of fresh polysaccharides,^78,88,89^ and for (cyclo)­hexanone, (cyclo)­pentanone, (alkyl)­furans, and (alkyl)­pyrans that are also products of carbohydrates and polysaccharides.^78,88,89^ PC2 thus represented carbohydrates that are rapidly degradable when applied to soil as inputs.^37^ PC3′s positive side represented rapidly degradable N-containing OM,^37^ as positive loadings were found for specific pyrolytic product of proteins (diketopiperazines)^90^ and chlorophyll (pristine, phytene, and phytadiene)^81^ together with phenols, aromatic N compounds, (alkyl)­pyridines, and (alkyl)­pyrroles, i.e., pyrolytic and degradation products of proteins and chlorophylls (Figure 1a).^76,77,91^ Finally, PC4 separated lignin compounds (positive side), which are specific to higher plants and not rapidly degradable when applied to soils as inputs,^37^ from lipid-derived alkanoic acids and steroids (negative side), which are degradable in aerobic conditions but less degradable than proteins and carbohydrates.^86,92^ Altogether, the Py-GC/MS data set highlights five main OM composition types within the investigated organic inputs in terms of OM biochemical classes (e.g., carbohydrates versus N compounds) and degradation status.

**Figure 1:**
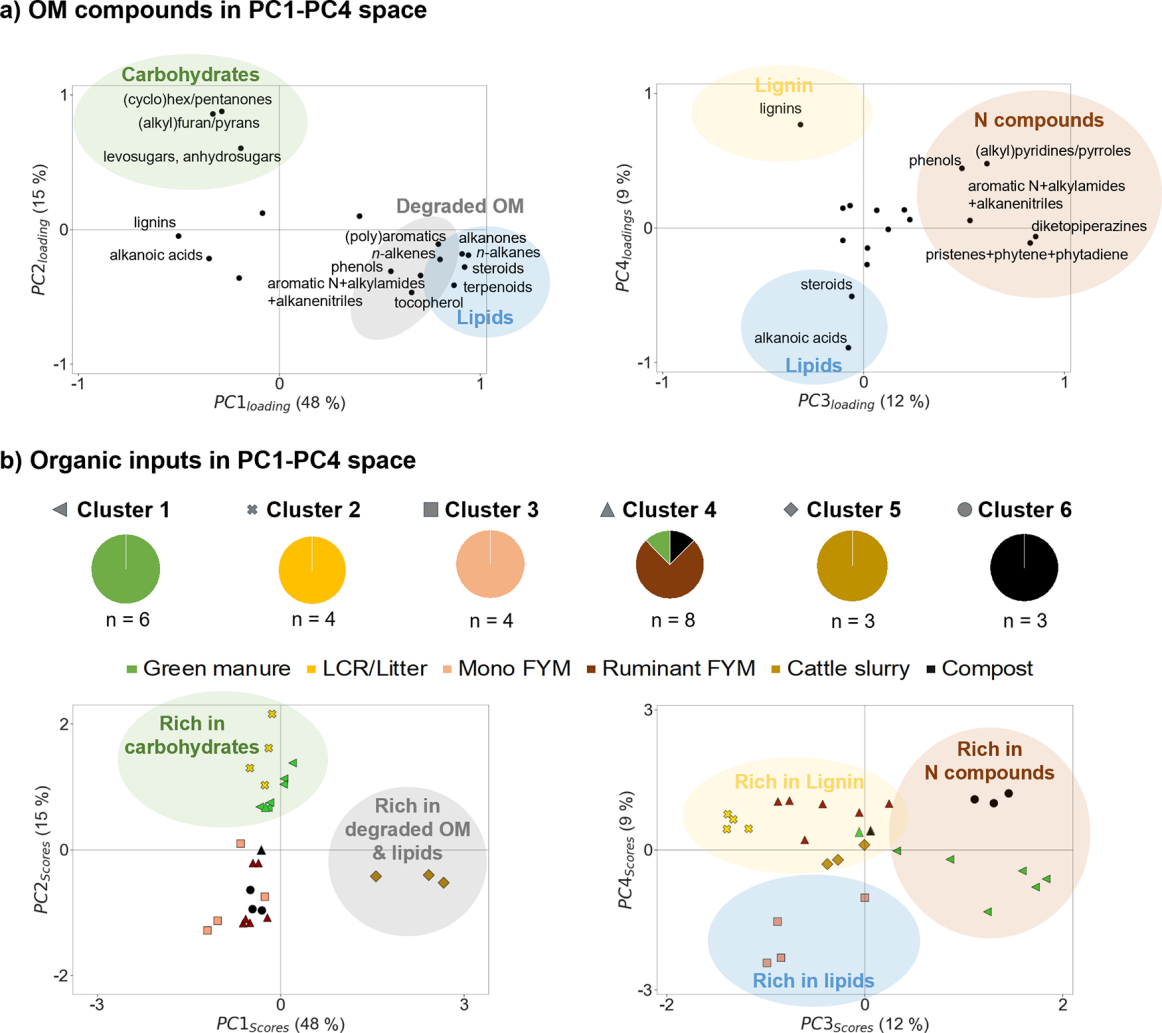
Differences in OM molecular composition across organic inputs as highlighted by principal component analysis (PCA) and hierarchical cluster analysis performed using the Py-GC/MS data set. Panel (a) shows the loadings of the 17 identified organic compound groups for the four extracted principal components, which captured 48% (PC1), 15% (PC2), 12% (PC3), and 9% (PC4) of total variance (in total, 84% of total variance). All compound groups are shown with black dots, but only the compound groups that had a PC-loadings value >0.39 (i.e., *R* ^2^ > 0.5) are labeled. *n-*Alkanes include chain lengths from C13 to C35, while *n*-alkenes include chain lengths from C9 to C33. Alkanoic acids include chain lengths from C4 to C31. Panel (b) shows the scores of the organic input samples (*n* = 28) on PCs 1–4. In this plot, marker shapes indicate clusters 1–6 determined using hierarchical cluster analysis (SPSS Statistics 23). Pie charts describe the organic input membership in each cluster. The organic input type “Lignified crop residues and litter” is abbreviated as “LCR/Litter” and “farmyard manure” is abbreviated as “FYM”.

### OM Molecular Composition Is Strongly Related to Organic Input Types

The 28 organic inputs were distributed far across PCs 1–4 ordination spaces, indicating a large variation in OM molecular composition (Figure 1b). The hierarchical cluster analysis performed on this data set (with a selection of six clusters) further demonstrates that the difference in OM composition closely follows the organic input type (Figure 1b and Table S10). All green manure samples (except from yellow mustard) were grouped in cluster 1, and all LCR/Litter samples were grouped in cluster 2. These clusters were enriched in carbohydrates (PC2 positive scores; Figure 1b) which, on average, accounted for 29 ± 4 and 39 ± 7% of total identified peak area in clusters 1 and 2, respectively (Table S9). Cluster 1 was also rich in rapidly degradable N-containing OM (PC3 positive scores) with proteins and chlorophyll accounting for 4 ± 1% and 7 ± 2%, respectively (Table S9). In contrast, LCR/Litter were depleted in N-containing compounds (total N compounds, 1.4 ± 0.4%) and rich in lignin (positive scores on PC4), which accounted for 50 ± 7% (Table S9). From these results and in agreement with a previous study,^40^ we can expect that the OM of the sampled green manures will be rapidly degraded when used as an organic input, with the exception of yellow mustard as it is grouped in cluster 4. The OM of LCR/Litter also contained OM that can degrade rapidly upon application to soils, but to a lesser extent than green manures due to high proportions of lignin that are not rapidly degradable compounds when applied to soil as inputs.^37^

Cluster 3 grouped all monogastric FYM and was dominated by rapidly degradable lipids (negative score on PC4), with the average relative abundance of alkanoic acids plus steroids reaching 61 ± 21% (Table S9). Cluster 3 was, however, depleted in rapidly degradable carbohydrates (levosugars, anhydrosugars; 1.8 ± 0.4), proteins (1.0 ± 1.5%), and chlorophyll (0.9 ± 0.5%). These results suggest that monogastric FYM may undergo degradation once applied to soils, but the speed and extent of degradation will likely be less than those produced with green manures, which are enriched in carbohydrates, proteins, and chlorophyll.

In contrast to monogastric FYM, ruminant FYM inputs (all found in cluster 4) were dominated by lignin and depleted in rapidly degradable lipids, i.e., alkanoic acids plus steroids (positive scores on PC4; Figure 1b). Lignin accounted for 55 ± 10% of these organic inputs (Table S9). Note that cluster 4 also included the green manure sample “yellow mustard” and the one-week-old compost, likely because of their enrichment in lignin but depletion in carbohydrates (in contrast to clusters 1–2). Cattle slurry samples, which all grouped in cluster 5, also differed from monogastric FYM (cluster 3). They plotted on PC1′s positive side, indicating they were enriched in degraded OM (e.g., (poly)­aromatic, aromatic N), biomacromolecules, and different types of lipids. These lipids included rapidly degradable lipids (alkanoic acids and steroids), but also cell wall or wax lipids (e.g., from straw) detected as *n-*alkanes and *n*-alkenes by Py-GC/MS.^80,82,83^ This enrichment in degraded OM and OM resistant to degradation (biomacromolecules and cell wall and wax lipids)^65,76–84^ is likely due to OM degradation in the anaerobic environment of the slurry storage tank, which houses both liquid waste and solid fecal matter.

Cluster 6 (mature composts) was enriched in N compounds (positive scores on PC3), likely derived from microbial biomass,^93^ as well as in lignin (positive scores on PC4), likely derived from inputs used to make the composts (e.g., green waste, woody waste). Cluster 6 contained the highest abundance in N-containing compounds (13 ± 1% versus 0.9–12% for the other inputs), but interestingly, they had a low N freshness index value, i.e., 19 ± 3% versus 35–81% in cluster 1—green manures—and 3–102% in all other input types (Table S9). This suggests a degraded status of the N-compound fraction in composts compared to other organic input types, especially green manures, in line with OM degradation during the aerobic composting process.^72^

Overall, our results demonstrate that OM molecular composition strongly depends on the organic input type. While ruminant FYM, cattle slurries, and composts are more enriched in both degraded OM and aliphatic OM resistant to degradation, green manures, LCR/Litter, and monogastric FYM contain higher proportions of N compounds, polysaccharides, and lipids like steroids that degrade more rapidly when added to soils, thus releasing low-molecular-weight organic compounds. Therefore, the application of these latter types of organic inputs could hold more potential to increase mobile and plant-available Zn and Cd through solubilization of soil Zn and Cd by complexation with low-molecular-weight DOM compounds released during OM degradation. Interestingly, Boiteau et al. found that numerous N-containing low-molecular-weight metabolites dominated the speciation of Zn and other divalent metals (e.g., Cu) in the water-soluble phase of grassland soils.^94^ In addition, Soltani et al. showed that the type of preceding crop influences Zn uptake by wheat with a relationship between water-soluble amino acids concentrations in amended soils and Zn concentrations in wheat grain.^8^ Green manure could thus be an optimal organic input candidate to increase Zn uptake by plants given its higher proportions of proteins (Table S9) and N compounds (Figure 1b). These properties favor the release of amino acids and other low-molecular-weight N compounds that can increase the plant availability of Zn in soils through complexation. However, this would not be ideal in soil with high Cd, as these complexation processes could also increase soil availability of Cd.

### Size and Multielement Characterization of Zn and Cd in Water Extracts

Water-soluble Zn and Cd accounted for 0.7–89 and 0.4–79% of total Zn and Cd concentrations, respectively (Table S11). Although accounting for a small fraction in some organic inputs, this pool is relevant for plant availability because it is released into the soil solution upon input application.^21,23^ The proportions of water-soluble Zn and Cd correlated positively (*r* ^2^ = 0.81; *p* < 0.0001), indicating a similar solubility of Zn and Cd in the investigated organic inputs.

To evaluate differences in water-soluble Zn and Cd speciation among organic inputs, we first employed SEC-UV-ICP-MS/MS. To the best of our knowledge, SEC-ICP-MS has been applied to only few compost samples to characterize speciation of elements in organic inputs.^54,95^ The SEC conditions we used were optimized previously to obtain the best chromatographic resolution and recoveries of trace elements in extracts targeting the water-soluble and OM fractions of soils.^50^ The chosen conditions separate and elute compounds and nanoparticles (up to ∼100 kDa or ∼40 nm) according to their size as well as charges (see Note S3, Figure S2, and Tolu et al.^50^). Free Zn^2+^ and Cd^2+^ did not elute from the SEC column under the employed operating conditions; hence, these species were quantified using WHAM modeling as presented in next section. Yet the sum of SEC peak areas for each element correlated positively with the element concentration in the extracts (*p* < 0.001–0.011; Figure S3), indicating the semiquantitative nature of our SEC-UV-ICP-MS/MS data. Thus, SEC-UV-ICP-MS/MS analysis provides complementary data to the WHAM modeling by providing characterization of Zn and Cd bound to DOM and small nanoparticles.

From the combination of multiple parameters offered by this method, i.e., size and charge (from order of elution), aromaticity (UV absorbance), and multielement detection (C, Fe, P, S, Zn, and Cd), we classified Zn and Cd into three size and chemical fractions. This classification is illustrated for a green manure and a compost sample in Figure 2a,b, with an overview of all samples in Figure 2c. The SEC chromatograms for all samples are provided in Figures S4 and S5.

**Figure 2:**
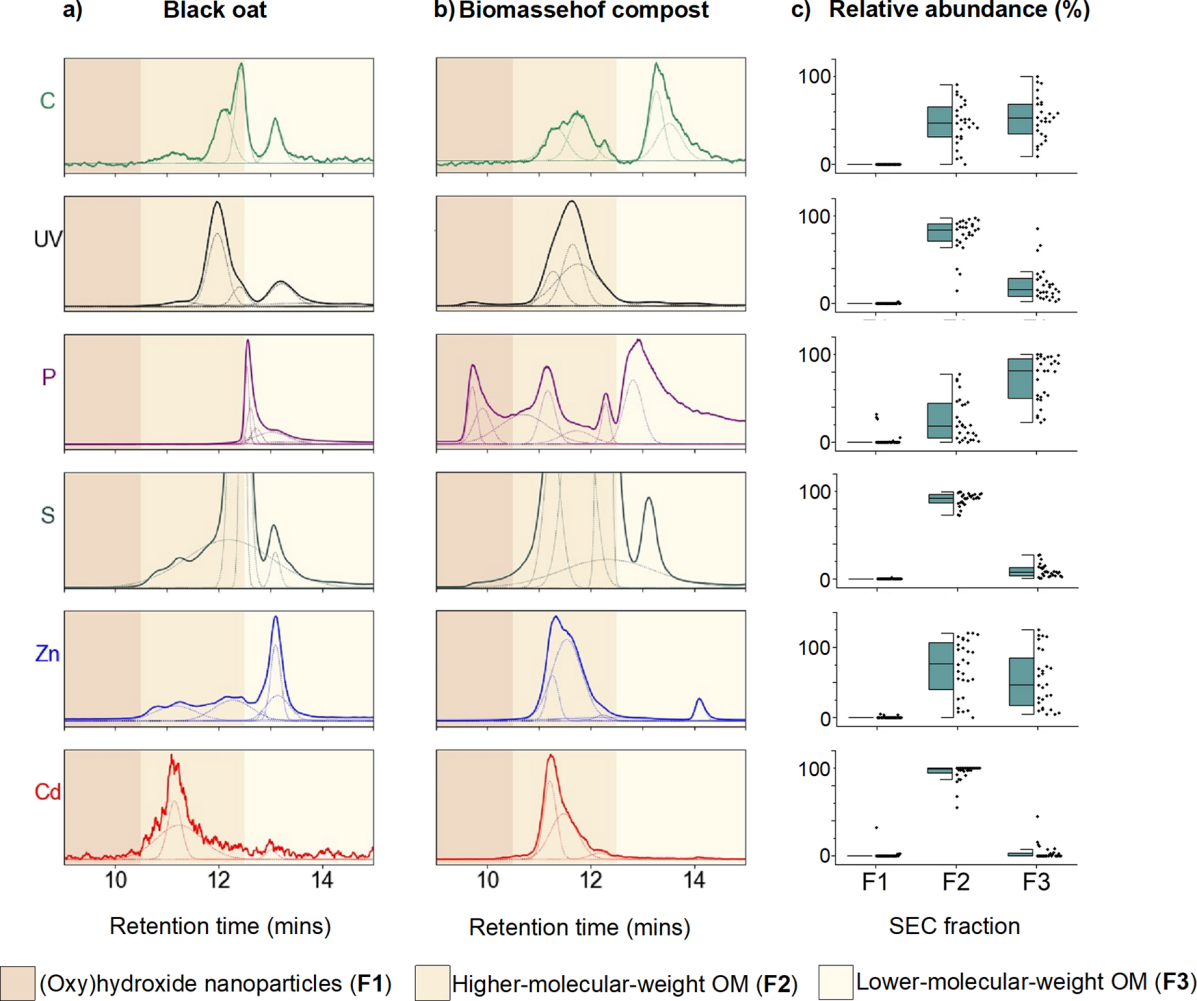
Organic input chromatograms and relative abundances of water-soluble carbon (C), ultraviolet absorbance (UV), phosphorus (P), sulfur (S), zinc (Zn), and cadmium (Cd) in the three size and chemical fractions detected by SEC-UV-ICP-MS/MS. Panel (a) provides UV and elemental chromatograms of black oat water extract (green manure), and panel (b) provides UV and elemental chromatograms of Biomassehof compost water extract (compost). Dotted lines show peak deconvolutions with OriginPro 2021. Panel shadings indicate ranges for peak apexes in fractions F1, F2, and F3. Panel (c) provides half boxplots calculated from the relative abundance of UV and elements in each fraction (F1, F2, and F3) for all organic inputs sampled (*n* = 28). The boxplots (made using OriginPro 2021) show the interquartile range, which represents the middle 50% of the data and falls between the upper quartile (75% data below that score) and the lower quartile (<25% below that score). The whiskers refer to the 5th/95th percentiles, the line indicates the median value, and black dots provide the values for all samples.

The first fraction (F1) corresponded to Fe and Mn peaks, which were the first eluting SEC peaks considering all elements (peak apex at 9.5–10.5 min; Figure S6). Fraction F1 was not associated with UV and C signals (Figures 2c and S6) and eluted before the UV peaks of reference organic materials (Figure S2). F1 thus represents (oxy) hydroxide nanoparticles, as observed for soils,^50^ and was a very minor fraction in the sampled organic inputs when present (Figure 2c). Fraction F2 was defined by the first eluting UV peaks, which were coeluting with peaks of C, S, Fe, and P as well as Zn and Cd (peak apex between 10.5 and 12.5 min; Figures 2a,b and S7). All UV and element signals observed for reference organic materials also eluted during this time range (Figure S2). F2 thus represents higher-molecular-weight and negatively charged OM that included aromatic OM rich in phenolic and carboxylic acid groups that are well known to contain or bind Fe, S, and P (e.g., proteins, lignins, phenols).^96–101^ Note that the large, thin peak of S eluting at 12.2 min in F2 corresponds to sulfate (Figure S8), which is due to its negative charge and thus earlier elution by negative charge repulsion from the column.^50^ Therefore, contribution of S to the organic fraction F2 is overestimated in Figure 2c. The third fraction (F3) consisted of C peaks coeluting with P, S, Zn and, for some samples with UV and Cd peak, with retention time longer than 12.5 min (Figures 2a,b and S7). These element and UV peaks eluted after the UV and element signals were detected for reference humic and fulvic acid materials (Figures 2a,b and S7). F3 was thus assigned as lower-molecular-weight, less negatively charged, hydrophilic OM, in line with previous studies showing the late elution of small hydrophilic OM compounds not absorbing much UV-light.^102,103^ Interestingly, the signal of UV, S, Fe, and Cd was predominantly associated with SEC fraction F2 in all investigated organic inputs, while the signal of C, P, and Zn was distributed across fractions F2 and F3 (Figure 2c). This highlights the difference in Zn association with DOM across organic inputs compared to Cd, which is discussed thereafter by combining the SEC-UV-ICP-MS/MS data set with WHAM modeling of Zn and Cd speciation.

### Distribution between Inorganic and DOM-Bound Zn and Cd in Water Extracts

In addition to SEC-UV-ICP-MS/MS characterization of water-soluble Zn and Cd, geochemical equilibrium modeling (WHAM VII) was used to estimate the distribution between inorganic species (free Zn^2+^, Cd^2+^, and their inorganic complexes) and DOM-bound forms. Due to the heterogeneity of organic inputs in terms of OM composition, modeling was performed using different “reactive DOM” values, which represent the proportion of DOM with high metal binding affinity (fulvic acid-like DOM)—typically estimated at 50–65% in soil and aquatic systems.^59,63^ Figure 3 compares Zn-DOM and Cd-DOM proportions modeled with reactive DOM values of 10, 50, 65, and 90%, and a SEC-based reactive DOM value (“SEC%”), derived from dividing the peak area of C detected in fraction 2 “Higher-molecular-weight, humic- and fulvic-like OM” by the sum of C SEC peak area. Across most inputs, Zn-DOM proportions were highly similar for reactive DOM values set to ≥ 50% and based on SEC data but significantly lower at 10% (Figure 3a). An exception was cattle slurries, where Zn-DOM proportions modeled using SEC-based reactive DOM matched those modeled with 10% of reactive DOM. The proportions of Cd-DOM were generally lower than those of Zn-DOM, except in green manures, and showed similar trends across the different organic inputs (Figure 3b).

**Figure 3:**
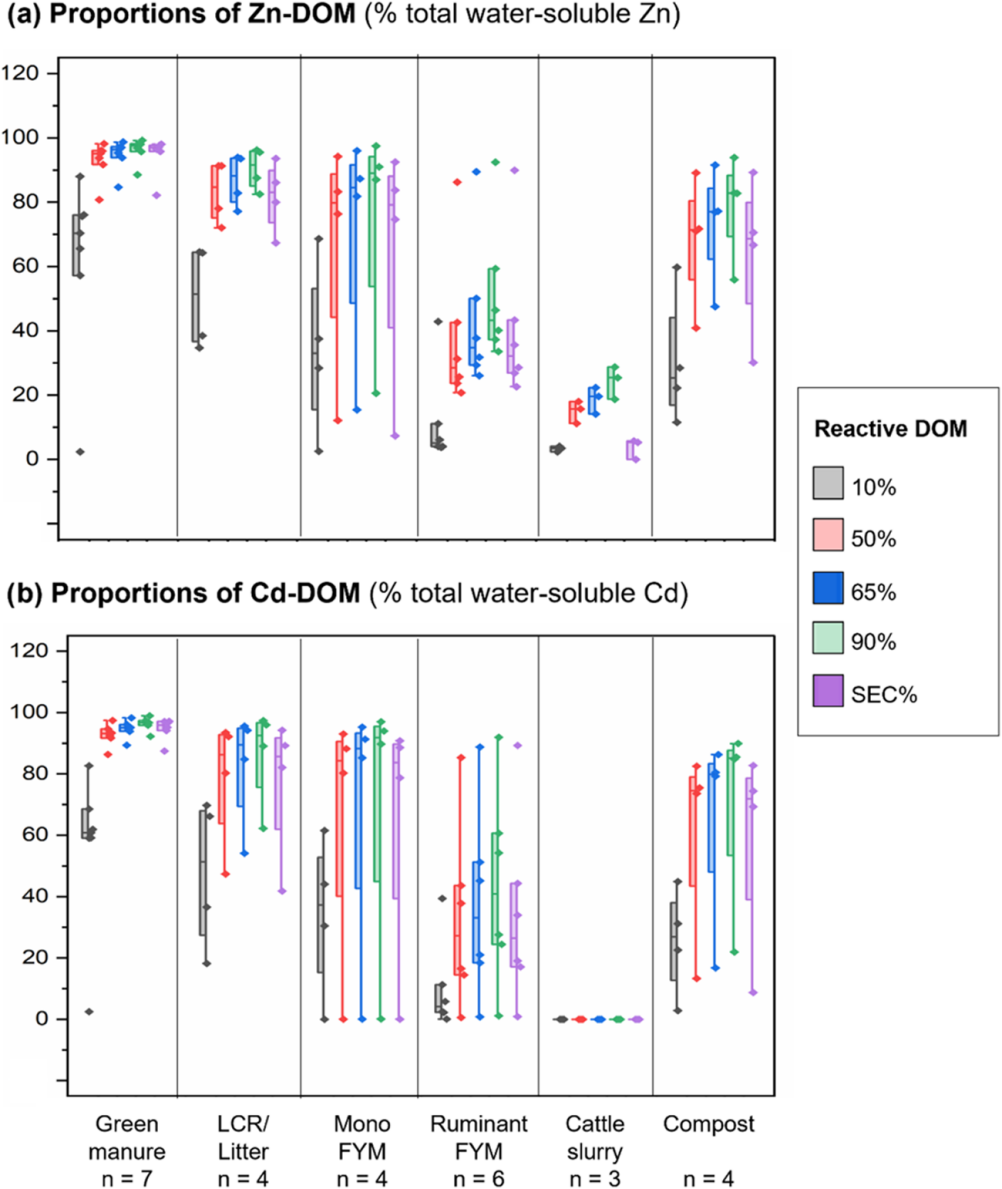
Proportions of water-soluble Zn and Cd bound to dissolved organic matter (DOM) modeled using WHAM VII and different values for the input parameter “reactive DOM”. The modeled proportions of Zn-DOM (panel (a)) and Cd-DOM (panel (b)) are shown as half boxplots with the organic inputs grouped according to their source types, which closely follows their OM composition (see Figure 1b). The boxplots (made with OriginPro 2021) show the interquartile range, which represents the middle 50% of the data and falls between the upper quartile (75% data below that score) and the lower quartile (<25% below that score). The whiskers refer to the 5th/95th percentiles, and the line indicates the median value. The values used for the input parameter “reactive DOM” (i.e., DOM reactive for complexation with trace metals like Zn and Cd) were 10, 50, 65, and 90% or were estimated based on the C data obtained with SEC-UV-ICP-MS/MS (labeled “SEC%”). This latter reactive DOM value was determined by dividing the peak area of C detected in fraction 2 “Higher-molecular-weight, humic- and fulvic-like OM” by the sum of area of detected C peaks and was of 63 ± 29% (range, 6–91, *n* = 7) for green manures, 48 ± 15% (range, 31–65, *n* = 4) for LCR/Litter, 42 ± 9% (range, 29–51, *n* = 4) for monogastric FYM, 59 ± 11% (range, 43–73, *n* = 6) for ruminant FYM, 13 ± 12% (range, 0–25, *n* = 3) for cattle slurries, and 43 ± 8% (range, 32–51, *n* = 4) for composts.

Zn-DOM and Cd-DOM dominated the water-soluble fractions (>60%) in green manures, and most LCR/litter and monogastric FYM. This was also the case for most compost samples when considering reactive DOM values >50%, which is expected based on previous studies.^64^ In contrast, in cattle slurry samples, inorganic Zn and Cd species (i.e., free Zn^2+^ and Cd^2+^ and aqueous inorganic complexes) accounted for >70% of the water-soluble pool (Zn-DOM and Cd-DOM < 30%). Among FYM samples, Zn-DOM and Cd-DOM proportions varied widely. Three of four monogastric FYM samples resembled green manures and composts, while the monogastric FYM sample “pig slurry” resembled cattle slurry (Zn-DOM and Cd-DOM < 30%). The Zn-DOM and Cd-DOM proportions of the six ruminant FYM also ranged between the low proportions found in cattle slurries and the higher proportions found in green manures, LCR/Litter and composts, particularly when modeled with reactive DOM values >50%. Overall, these findings underscore the critical role of the “reactive DOM” input parameter in WHAM-based modeling of Zn and Cd speciation in organic inputs, which is consistent with observations of Zn and other elements in aquatic systems and/or soils, e.g., Mueller et al. and Djae et al.^104,105^ While the choice of reactive DOM value influences model outcomes, our results highlight the dominant role of DOM in controlling water-soluble Zn and Cd speciation in green manures, LCR/litter, monogastric FYM, and composts and to a (much) lesser extent in ruminant FYM and cattle slurries.

### Water-Soluble Zn and Cd Speciation Differs between More Plant- and Animal-Waste-Based Organic Inputs

To compare the obtained Zn and Cd speciation results across all organic inputs, hierarchical cluster analysis was conducted using both SEC-UV-ICP-MS/MS data (Zn and Cd distribution among F2 and F3, expressed as % of total peak area) and WHAM modeling data (Zn and Cd partitioning between DOM-bound and inorganic forms, assuming 65% reactive DOM). Results showed that water-soluble Zn and Cd speciation varied less across organic input types than did OM molecular composition. Indeed, unlike the Py-GC/MS data set (Figure 2b), the inputs did not cluster by their origin (e.g., no clear separation between green manures, LCR/litter, monogastric FYM, ruminant FYM, cattle slurries, and composts; Table 2). Still, using four clusters, a distinction emerged between plant-based inputs (clusters 1–2) and animal-waste-based inputs (clusters 3–4; Table 2).

**Table 2:**
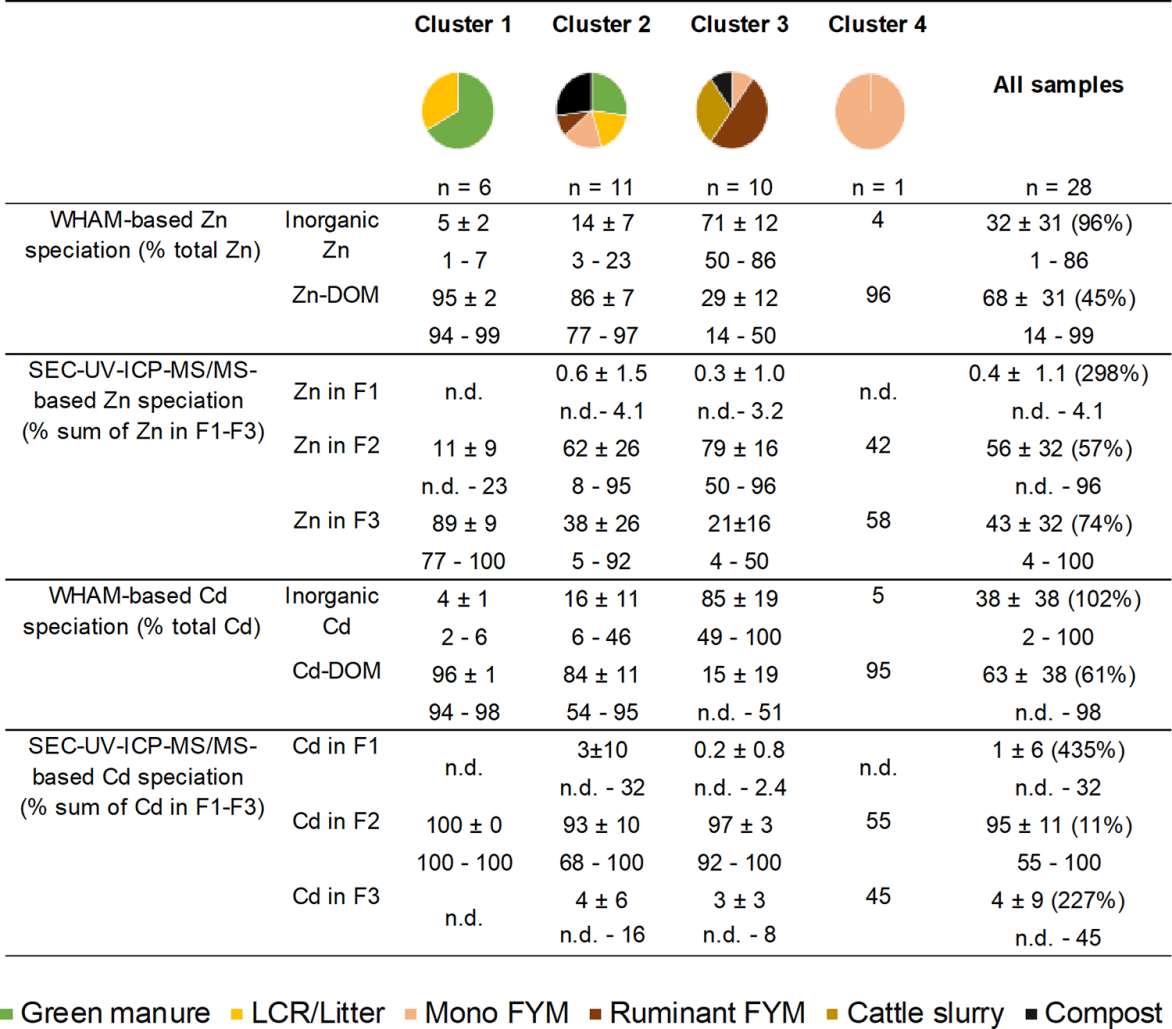
Water-Soluble Zn and Cd Speciation in the Investigated Organic Inputs Grouped According to the Cluster Analysis Performed Using Both WHAM-Based and SEC-UV-ICP-MS/MS-Based Data^a^

a For each parameter, the average and standard deviation (av ± 1 sd) as well as the minimal and maximal (min–max) values of each cluster and of the entire sample set are provided. For the entire sample set, the overall relative standard deviation (RSD, in %) is provided in parentheses after the average and standard deviation values. The WHAM-based Zn and Cd speciation data used were those obtained assuming a reactive dissolved organic matter (DOM) value of 65%. “Inorganic Zn” and “inorganic Cd” included Zn^2+^, ZnOH^+^, Zn­(OH)_2_, ZnSO_4_, ZnCO_3_, ZnCl^+^, ZnHCO_3_^+^ and Cd^2+^, CdOH^+^, Cd­(OH)_2_, CdSO_4_, CdCl^+^, CdCl_2_, CdHCO_3_^+^, CdCO_3_, Cd­(CO_3_)_2_^2–^, respectively. Values for “Zn-DOM” and “Cd-DOM” refer to WHAM modeling results for water-soluble Zn and Cd bound to DOM. For the SEC-UV-ICP-MS/MS-based data, F1 corresponded to “(oxy)­hydroxide nanoparticles”, F2 corresponded to “higher-molecular-weight OM”, and F3 corresponded to “lower-molecular-weight OM”. A detailed description of the size and chemical properties of SEC fractions F1-3 is provided in the above text, “Size and multi-element characterization of Zn and Cd in water extracts”. A detailed list of samples in each speciation cluster can be found in Table S12. The organic input type “lignified crop residues and litter” is abbreviated as “LCR/Litter,” “monogastric” is abbreviated as “Mono”, and “farmyard manure” is abbreviated as “FYM”. Zn and Cd SEC fractions that were not detected are indicated as “n.d.”

Cluster 1 (composed of green manures and LCR/Litter), cluster 2 (mixing green manures, LCR/Litter, composts, two monogastric FYM and one ruminant FYM), and cluster 4 (one monogastric FYM) were characterized by high proportions of Zn-DOM and Cd-DOM, i.e., >77%, except for one LCR/Litter (shredded corn) having 54% of Cd-DOM. In contrast, cluster 3, which grouped 5 of 6 ruminant FYM with all cattle slurries, one monogastric FYM (pig slurry), and one compost (Gerber compost 3; Table S12), showed higher proportions of inorganic Zn and Cd species (e.g., Zn^2+^, Cd^2+^, ZnOH^+^, CdOH^+^, ZnSO_4_, CdSO_4_). Interestingly, this may be linked to the pH and OM molecular composition. The higher pH of animal-based inputs (9.5 ± 1.0; Table 1, cluster 3) and of the Gerber compost 3 (9 versus ∼8 in other composts) may promote the formation of inorganic Zn and Cd complexes over DOM binding. Moreover, Py-GC/MS revealed that ruminant FYM and cattle slurries were richer in lignin and degraded and aliphatic OM, and poorer in carbohydrates and N compounds—functionalized OM types with higher Zn and Cd affinity (e.g., hydroxyl, amine, and thiol functional groups; Figure 1b and Table S9). All compost samples had a higher pH (8.4 ± 0.5; Table 1, cluster 4), similar to ruminant FYM and cattle slurries. But they were richer in N compounds and carbohydrates and showed higher levels of water-soluble Zn-DOM and Cd-DOM, thus clustering with green manures and LCR/litter based on speciation data (Table 2, cluster 2). These results suggest a key role of OM composition, beyond pH, in controlling water-soluble Zn and Cd partitioning between DOM-bound and inorganic forms in organic inputs.

Cluster 1 differed from clusters 2 and 4 by showing a higher proportion of Zn in SEC fraction F3—representing lower-molecular-weight, less negatively charged, hydrophilic, and P-rich OM (∼90% vs ≤ 50% of total Zn peak area). This greater Zn association with F3 in green manures and LCR/litter (Table 2) is consistent with Zn binding to organic P compounds in plants,^25,106–108^ as shown by synchrotron X-ray spectroscopy. In clusters 2 (mix of organic inputs), 3 (mostly animal waste inputs), and 4 (one monogastric FYM), Zn was more associated with higher-molecular-weight, negatively charged, S-rich OM (SEC fraction F2) than with SEC-F3, likely due to interactions with phenolic, carboxylic, and reduced S groups as also observed in plants.^27,109^ Cd showed a different pattern: clusters 1 and 2 had similarly high Cd proportions in F2 (100 and 93%, respectively). Across all samples, only one sample—a monogastric FYM (cluster 4)—had higher Cd in F3 (45% vs <16% in other samples). It is possible that lower water-soluble Cd concentrations in our samples (overall 20 ± 20 μg kg^–1^, ranging 0.01–77 μg kg^–1^) may have prevented F3 peaks from being detected, underestimating Cd association with lower-molecular-weight, less negatively charged, hydrophilic P-rich OM. This underscores the analytical limitations in resolving Cd speciation in organic inputs due to low concentrations. Previous studies showed using synchrotron-based X-ray spectroscopy that Cd binds to O-, N-, and reduced S-containing functional groups in plants, rice straw, and cacao tree branches^34,35,110^ while Zn binds to organic P compounds (like phytate).^25,106–108^ Cd also showed lower affinity and complex stability with phytate compared to Zn.^111^ These findings support the distinct binding of Zn and Cd to DOM in organic inputs, as revealed by our SEC-UV-ICP-MS/MS analysis.

Overall, our operationally defined, functional speciation data set reveals that water-soluble Zn and Cd speciation varies across organic inputs, with distinctions between plant-based and animal-waste-derived materials. Ruminant FYM, cattle slurry, and some monogastric FYM samples, which contained higher total Zn and Cd concentrations (Table 1, cluster 3), may directly increase the plant-available Zn and Cd pool due to their greater proportions of soluble free ions and inorganic complexes. In contrast, composts with similarly high Zn and Cd concentrations as animal waste inputs primarily contain water-soluble Zn and Cd bound to higher-molecular-weight DOM, which are less likely to be readily plant-available and more likely to be accumulated in soils.^112^ Similarly, green manures and LCR/litter predominantly contained water-soluble Cd bound to higher-molecular-weight DOM, suggesting a low risk of Cd availability. As plant-derived inputs were found to contain lower Cd concentrations than animal-waste-derived and compost inputs (Table 1, clusters 1–2), they appear to be associated with lower risks of Cd accumulation in soils. For Zn, the plant-based inputs (green manures and LCR/Litter) showed Zn associated primarily with lower-molecular-weight DOM (SEC-F3; Figure S5). Thus, further research is needed to clarify whether these lower-molecular-weight Zn-DOM complexes can be taken up by plants and how they vary among plant-based inputs (green manure and LCR/Litter).

### Environmental and Analytical Implications

As societies transition toward a circular economy to meet global food demands sustainably, e.g., by replacing synthetic fertilizers with organic inputs of biological origin, a comprehensive characterization of these materials becomes increasingly urgent.^12,113^ Our study provides the first systematic and detailed chemical characterization of a wide variety of organic inputs, offering an essential base for understanding their composition and variability. This knowledge can help interpret the effects of organic inputs on Zn and Cd plant availability versus accumulation in soils, thereby better predicting their fate in circular agroecosystems. For example, earlier studies showed that green manure from clover and sunflower increased Zn plant uptake, whereas mustard green manure did not.^10,75,114^ Our data may explain this: unlike the other analyzed green manures, yellow mustard manure was found to lack enrichment in N compounds and proteins and was enriched in lignin (found in cluster 4; Figure 1a). This would follow that yellow mustard green manure is less rapidly degraded in soils, producing less dissolved, low-molecular-weight compounds (e.g., organic acids, amino acids) that can complex Zn and thus lead to a release of soil Zn in soil solutions^29,115^ Future studies are, however, needed to explore how water-soluble Zn and Cd speciation change during storage or post-treatment of these materials. Also, pot and field experiments are ultimately required to determine which organic input types enhance crop Zn and/or Cd concentrations or lead to soil contamination since these outcomes also depend on soil properties, plant cultivar, and land management strategies. Our results can guide the prioritization of organic inputs that should be progressively tested in such time- and resource-intensive crop growth experiments in the context of Zn and Cd fluxes in circular and sustainable agroecosystems.

Our study presents the first speciation data for Zn in green manures and LCR/litter and the first for Cd in organic inputs. Although environmentally relevant, speciation here is operationally defined, and characterization of the chemical identity or binding environment of Zn and Cd remains to be done. Molecular elucidation of the water-soluble Zn-DOM and Cd-DOM complexes in organic inputs, especially those we detected in the lower-molecular-weight hydrophilic OM fractions, would improve predictions of their degradability and plant availability. To achieve this, analytical developments are needed and could build upon our SEC-UV-ICP-MS/MS speciation data and upon previous studies coupling SEC^102^ or other types of liquid chromatography^94^ to high-resolution mass spectrometry (HR-MS). Particularly, methods allowing preconcentration and/or further isolation of the different Zn-DOM and Cd-DOM complexes (while keeping their integrity) are needed to be able to detect and determine the chemical formulas of these compounds by HR-MS. This is due to the low total Zn and Cd concentrations in the water-soluble phase of organic inputs and the fact that Zn-DOM and Cd-DOM complexes likely include tens to hundreds of compounds given the SEC chromatograms we observed in this study. An approach could be to collect the SEC fractions in which we observed Zn and/or Cd and then preconcentrate them, e.g., by evaporation before analysis via reversed-phase chromatography coupled to HR-MS as performed in biological samples (e.g., studies of Aurelie et al. and Cvetkovic et al).^57,116^ In addition, future studies are needed to consider the contribution of nanosized ZnS and CdS to water-soluble speciation of organic inputs given that ZnS was found as an important species in the solid phase of organic biowaste.^19^ Finally, the high Zn proportions in P-rich DOM fractions, and the known presence and importance of Zn-phytate complexes in agricultural systems,^117^ also highlight the needs for method(s) enabling the quantification of phytate and phytate-Zn complexes in organic inputs.

## Supplementary Material



## Notes

Detailed sample descriptions and selection criteria; extended methods and quality control results; additional visualizations of Py-GC/MS data; SEC-UV-ICP-MS/MS data; and statistical analysis outputs (PDF)

## ∥

Department of Applied Microbial Ecology, Helmholtz Institute for Environmental Research (UFZ), Permoserstraße 15, 04318 Leipzig, Germany
