# Supplementary material for: Drastic Variations in Chemical Composition of Organic Inputs: Implications for Organic Fertilization
Source: Environ Sci Technol. 2025 Jul 28;59(31):16463–77. doi: 10.1021/acs.est.5c06493 (PMC12355952; doi:10.1021/acs.est.5c06493)
Supplement: Supplementary file 1 [file es5c06493_si_001.pdf]

Drastic variations in chemical composition of organic inputs: implications for organic fertilisation

Jill Bachelder[a,b,c,#] *, Matthias Wiggenhauser[c], Lenny H.E. Winkel[a,b], Emmanuel Frossard[c], Julie Tolu[a,b] *

[a]ETH Zurich, Swiss Federal Institute of Technology, Department of Environment Systems Sciences (D-USYS), Institute of Biogeochemistry and Pollutant Dynamics (IBP), Group of Inorganic Environmental Geochemistry, Universitätstrasse 16, 8092 Zurich, Switzerland

[b]Eawag, Swiss Federal Institute of Aquatic Science and Technology, Department of Water Resources and Drinking Water (W+T), Überlandstrasse 133, 8600 Dübendorf, Switzerland;

[c]ETH Zurich, Swiss Federal Institute of Technology, Department of Environment Systems Sciences (D-USYS), Institute of Agricultural Sciences (IAS), Group of Plant Nutrition, Eschikon 33, 8315 Lindau, Switzerland

Present addresses:
[#] Department of Applied Microbial Ecology, Helmholtz Institute for Environmental Research (UFZ), Permoserstraße 15, 04318 Leipzig, Germany

*Email: julietolu@hotmail.com;
* Email: jill.bachelder@ufz.de

Summary: 36 pages, 8 figures, 12 tables, 3 notes, 4 supplementary methods

Table of contents

PART I: SAMPLE INFORMATION AND METHODS S4

Table S1. Organic input sampling procedure, storage, and processed replicates S4

Note S1. Choice of sampled organic inputs S5

Note S2. How representative are our samples? S6

Method S1. Py-GC/MS analysis of organic inputs S7

Table S2. Detailed information on Py-GC/MS analysis of organic inputs S8

Table S3. List of pyrolytic products measured by Py-GC/MS analysis of organic inputs S10

Figure S1. Correlation between total carbon (C) concentrations and total identified peak areas from the Py-GC/MS analysis of organic inputs S15

Method S2. Digestion of organic inputs (solid and water-soluble pool) S15

Method S3. Quantification of total elemental concentrations by ICP-MS(/MS) S16

Table S4. Measured and certified elemental concentrations in solid samples S17

Table S5. Measured and certified elemental concentrations in liquid samples S18

Table S6. Solid-liquid (S:L) ratios used for water extractions of the organic inputs. S19

Method S4. SEC-UV-ICP-MS/MS analysis of organic input water extracts S20

PART II: SUPPLEMENT TO RESULTS AND DISCUSSION S21

Table S7. Output of the hierarchical cluster analysis performed on bulk properties. S21

Table S8. Summary of literature values for the total concentrations of Zn and Cd in organic inputs S22

Table S9. Biochemical class and organic matter (OM) molecular composition summary for six clusters based on the Py-GC/MS dataset S23

Table S10. Output of the hierarchical cluster analysis performed with the Py-GC/MS dataset S24

Table S11. Percentage of water-soluble elements in each cluster based on water-soluble Zn and Cd speciation S25

Note S3: Separation of trace element compounds by size exclusion chromatography S26

Figure S2. Molecular weight and humic standards analyzed with size exclusion chromatography S27

Figure S3. Correlation between the sum of element SEC peak area and total concentrations in the water extracts S28

Figure S4. Chromatograms showing Fe, UV, and C elution and intensity in all organic input samples S29

Figure S5. Chromatograms showing P, S, Zn, and Cd elution and intensity in all organic input samples S30

Figure S6. Definition of fraction 1 (F1) of SEC analyses in water extracts of organic inputs S31

Figure S7. Definition of fraction 2 (F2) and fraction 3 (F3) of SEC analyses in water extracts of organic inputs S32

Figure S8. UV and element chromatograms of a water blank measured by SEC-UV-ICP-MS/MS, provided in the following order from top to bottom S33

Table S12. Output of the hierarchical cluster analysis performed on WHAM-based and SEC-UV-ICP-MS/MS-based datasets S34

REFERENCES S35

Part I: Sample information and methods

Table S1. Organic input sampling procedure, storage, and processed replicates. The organic input type "Lignified crop residues and litter" is abbreviated as "LCR/Litter", while "farmyard manure" is abbreviated as "FYM". Green manure seeds were purchased from UFA Samen (www.ufasamen.ch). "Industrial compost" refers to "compost" in main manuscript and originated from aerobic composting of inputs that included manure, crop residues, and garden waste. More information can be found at the following links: Strickhof (www.strickhof.ch), FiBL (www.fibl.org), Agroscope (www.agroscope.admin.ch), Biomassehof AG (www.biomassehof.ch), and Gerber AG (www.gerber.ch). "Industrial compost" refers to "compost" in main manuscript.

Input type	Sample type	Sample name	Site (city)	Sampling procedure (age, if known)	No. process replicates
Green manure	Green manure	Berseem clover	Strickhof (Lindau)	Above-ground biomass sampled from field	2
	Green manure	Black oat	Strickhof (Lindau)	Above-ground biomass sampled from field	2
	Green manure	Phacelia	Strickhof (Lindau)	Above-ground biomass sampled from field	1
	Green manure	Subterranean clover	Strickhof (Lindau)	Above-ground biomass sampled from field	1
	Green manure	Yellow mustard	Strickhof (Lindau)	Above-ground biomass sampled from field	3
	Green manure	Green manure mix 1	Strickhof (Lindau)	Above-ground biomass sampled from field. Included berseem clover (100 g), persian clover (60 g), phacelia (40 g)	1
	Green manure	Green manure mix 2	Strickhof (Lindau)	Above-ground biomass sampled from field. Included berseem clover (60 g), summer vetches (190 g), phacelia (40 g), and Guizotia (10 g)	2
LCR/Litter	LCR	Barley straw	Strickhof (Lindau)	From barn	1
	LCR	Shredded corn	Strickhof (Lindau)	Patch of 1 m^2 direct from field	3
	LCR	Wheat straw	Strickhof (Lindau)	From barn	1
	Litter (bedding, <30% manure)	Poultry litter	FiBL (Frick)	From stall	3
Monogastric FYM	FYM	Pig manure (fresh)	Strickhof (Lindau)	Fresh from back end	1
	FYM	Pig manure (pile)	Strickhof (Lindau)	From pile (<1 week old)	3
	Slurry	Pig slurry	Strickhof (Lindau)	From under stall (<1 week old)	2
	FYM	Poultry manure	Strickhof (Lindau)	From pile (<2 weeks old)	3
Ruminant FYM	FYM	Cattle manure	Agroscope (Zurich)	From pile (<1 month old)	1
	FYM	Horse manure	Strickhof (Lindau)	From pile (<1 month old)	3
	FYM	Mixed manure pile	Strickhof (Lindau)	From pile (<1 month old)	4
	Compost	Composted cattle manure 1	DOK Trial (Therwil)	From pile (2-3 months old, 4 weeks composted with biodynamic treatment)	3
	Compost	Composted cattle manure 2	DOK Trial (Therwil)	From pile (2-3 months old, 4 weeks composted with bioorganic treatment)	3
	Compost	Composted catte manure 3	DOK Trial (Therwil)	From pile (2-3 months old, 4 weeks composted)	3
Cattle slurry	Cattle slurry	Cattle slurry 1	DOK Trial (Therwil)	Sampled from tap at bottom of tank without prior mixing (<1 month old). Biodynamic treatment.	2
	Cattle slurry	Cattle slurry 2	FiBL (Frick)	Sampled upper half of storage tank without prior mixing. Biodynamic treatment.	2
	Cattle slurry	Cattle slurry 3	FiBL (Frick)	Sampled upper half of storage tank without prior mixing.	2
Industrial compost	Compost	Gerber compost 1	Gerber Bio Greens AG (Fehraltorf)	From pile (after 1 week of composting)	2
	Compost	Gerber compost 2	Gerber Bio Greens AG (Fehraltorf)	From pile (after 4 weeks of composting)	2
	Compost	Gerber compost 3	Gerber Bio Greens AG (Fehraltorf)	From pile (after 8 weeks of composting)	6
	Compost	Biomassehof compost	Bio Massehof AG (Winterthur)	From pile (after 3 months of composting)	1

Note S1. Choice of sampled organic inputs

Our aim was to sample organic inputs of highly diverse chemical composition and to capture the variability in their OM composition and Zn and Cd speciation. Some samples were intentionally included in this study because they provided insight into the variability in organic input chemical composition. For example, we chose to include both poultry manure from a pile and poultry litter (wood chip bedding + <30 % manure). This was because we wanted to understand how OM composition and Zn and Cd speciation would differ due to the relatively low amount of manure and high amount of bedding in poultry litter compared to manure collected from a pile. We also included pig manure from a pile and pig manure fresh from the backend (i.e., a fresh turd). This allowed us to evaluate the degree to which the amount of bedding and manure freshness would impact the OM molecular composition of the inputs. Similarly, we sampled compost from Gerber AG after 1, 4, and 8 weeks of composting, to observe the effect of composting time on our results (even though the compost is only made commercially available after at least 8 weeks of composting). Thus, some samples were selected to provide insight into variation in composition due to factors such as composting time, ratio of bedding to manure, and manure freshness.

Note S2. How representative are our samples?

Though we have done our best to sample a wide range of organic inputs, our dataset is not all-encompassing. The organic inputs presented in this study were sampled in a few regions of Switzerland, so differences in chemical composition related to vastly different land management practices were not captured. Firstly, no manure was sampled from intensive conventional farms. This could be one reason why the sampled inputs did not contain high concentrations of Zn and Cd compared to some literature values (Table S8). Secondly, as none of the cattle slurry samples in this study were actively mixed (homogenized) immediately before sampling, none of the cattle slurry samples can be considered as fully representative of the original slurry chemical composition. Nevertheless, our chemical characterization of cattle slurry does represent the broad range in potential composition (i.e., both high and low ratios of solid particulate matter to liquid waste). This is because we sampled two cattle slurries from the upper section of a storage tank (i.e., more liquid and less particulate matter; cattle slurry samples were “biodyn” and “no treatment”) that is aerated regularly and one slurry from the bottom section of a storage tank (i.e., more particulate matter; cattle slurry sample was “biodyn, DOK”) that is not aerated regularly. Thirdly, the green manure samples were all grown on a single field. This means that our study only captures variation in composition between green manure plant species and not between green manures grown in soil with highly varied Zn and Cd availability (which can affect total Zn and Cd concentrations in the green manures). Finally, several important categories of organic inputs were completely lacking from this study, including anaerobic digestates, anaerobically prepared composts (compogas), and monogastric farmyard manure (e.g., pig and poultry manure) stored for longer than 1 month. These organic inputs could have highly different OM molecular composition as well as Zn and Cd speciation as compared to the organic inputs sampled. For example, if we had included pig or poultry manure that had undergone long-term storage (e.g., longer than 1 month), it is possible we would have found that the samples to be depleted in rapidly degradable organic matter (as rapidly degradable compounds could be degraded by microbes during manure storage).

Method S1. Py-GC/MS analysis of organic inputs

Organic input samples were pyrolyzed at 450°C in an oven pyrolyzer (PY-2020iD, Frontier Labs) connected to an Agilent 7890A-5975C GC/MS. To inject a similar C amount, varied sample mass (100-500 µg) and injection split ratios (1/16 or 1/8; Table S2) were used to analyze the organic inputs. This was determined based on total C concentrations. The Py-GC/MS data was analyzed using the data processing method developed by Gerber et al.[1] Peak identification was performed using the NIST MS Search 2 software using the NIST/EPA/NIH 2011 library and published mass spectra.[2] Due to large differences in Py-GC/MS chromatograms between organic inputs, the data were processed in eight batches (Table S2) based on the total ion current chromatogram to maximize the number of peaks detected by the data-processing pipeline.

Table S2. Detailed information on Py-GC/MS analysis of organic inputs. Measurement involved two runs and eight data-processing batches for identification of pyrolytic products. For samples in “run 1”, a sample mass of 100-200 µg was analyzed using a split ratio of 1/16. For samples in “run 2”, a sample mass of 300-500 µg was analyzed using a split ratio of 1/8. “Total No. replicates” refers to the total number of replicates analyzed, including process replicates for samples processed with multiple process replicates (Table S1). “Industrial compost” refers to “compost” in main manuscript.

Input type	Sample name	Total No. replicates	Py-GC/MS Run No.	Py-GC/MS analysis settings	Data analysis batch
Green manure	Berseem clover	3	Run 1	100-200 µg of sample analyzed; split ratio of 1:16 used	Batch 1
	Black oat	3	Run 1	100-200 µg of sample analyzed; split ratio of 1:16 used	Batch 1
	Phacelia	3	Run 1	100-200 µg of sample analyzed; split ratio of 1:16 used	Batch 1
	Subterranean clover	3	Run 1	100-200 µg of sample analyzed; split ratio of 1:16 used	Batch 6
	Yellow mustard	3	Run 1	100-200 µg of sample analyzed; split ratio of 1:16 used	Batch 2
	Green manure mix 1	3	Run 1	100-200 µg of sample analyzed; split ratio of 1:16 used	Batch 1
	Green manure mix 2	3	Run 1	100-200 µg of sample analyzed; split ratio of 1:16 used	Batch 1
LCR/Litter	Barley straw	3	Run 1	100-200 µg of sample analyzed; split ratio of 1:16 used	Batch 2
	Shredded corn	3	Run 1	100-200 µg of sample analyzed; split ratio of 1:16 used	Batch 2
	Wheat straw	3	Run 1	100-200 µg of sample analyzed; split ratio of 1:16 used	Batch 2
	Poultry litter	3	Run 1	100-200 µg of sample analyzed; split ratio of 1:16 used	Batch 2
Monogastric FYM	Pig manure (fresh)	3	Run 1	100-200 µg of sample analyzed; split ratio of 1:16 used	Batch 5
	Pig manure (pile)	3	Run 1	100-200 µg of sample analyzed; split ratio of 1:16 used	Batch 5
	Pig slurry	3	Run 1	100-200 µg of sample analyzed; split ratio of 1:16 used	Batch 6
	Poultry manure	3	Run 1	100-200 µg of sample analyzed; split ratio of 1:16 used	Batch 1

Table S2 (continued). Detailed information on Py-GC/MS analysis of organic inputs. Measurement involved two runs and eight data-processing batches for identification of pyrolytic products. For samples in "run 1", a sample mass of 100-200 µg was analyzed using a split ratio of 1/16. For samples in "run 2", a sample mass of 300-500 µg was analyzed using a split ratio of 1/8. "Total No. replicates" refers to the total number of replicates analyzed, including process replicates for samples processed with multiple process replicates (Table S1). "Industrial compost" here refers to "compost" in main manuscript.

Classification	Sample ID	Total No. replicates	Py-GC/MS Run No.	Py-GC/MS analysis settings	Data analysis batch
Ruminant FYM	Cattle manure	3	Run 2	300-500 µg of sample analyzed; split ratio of 1:8 used	Batch 8
	Horse manure	6	Run 1	100-200 µg of sample analyzed; split ratio of 1:16 used	Batch 5
	Mixed manure pile	3	Run 1	100-200 µg of sample analyzed; split ratio of 1:16 used	Batch 1
	Mixed manure pile	1	Run 1	100-200 µg of sample analyzed; split ratio of 1:16 used	Batch 2
	Composted cattle manure 1	3	Run 1	100-200 µg of sample analyzed; split ratio of 1:16 used	Batch 3
	Composted cattle manure 2	3	Run 1	100-200 µg of sample analyzed; split ratio of 1:16 used	Batch 3
	Composted catte manure 3 (replicate 1)	3	Run 1	100-200 µg of sample analyzed; split ratio of 1:16 used	Batch 3
	Composted catte manure (replicate 2)	3	Run 1	100-200 µg of sample analyzed; split ratio of 1:16 used	Batch 3
Ruminant (cattle) slurry	Cattle slurry 1	3	Run 1	100-200 µg of sample analyzed; split ratio of 1:16 used	Batch 4
	Cattle slurry 2	3	Run 1	100-200 µg of sample analyzed; split ratio of 1:16 used	Batch 4
	Cattle slurry 3	6	Run 1	100-200 µg of sample analyzed; split ratio of 1:16 used	Batch 4
Industrial compost	Gerber compost 1	3	Run 2	300-500 µg of sample analyzed; split ratio of 1:8 used	Batch 8
	Gerber compost 2	3	Run 2	300-500 µg of sample analyzed; split ratio of 1:8 used	Batch 7
	Gerber compost 3	6	Run 2	300-500 µg of sample analyzed; split ratio of 1:8 used	Batch 7
	Biomassehof compost	6	Run 2	300-500 µg of sample analyzed; split ratio of 1:8 used	Batch 7

Table S3. List of pyrolytic products measured by Py-GC/MS analysis of organic inputs

Name	Ref.Mass spectra	Compounds groups
**Carbohydrates**		
Methyl-cyclopentanone	NIST	(cyclo)hex/pentanone
Dimethyl-cyclopentenone	NIST	
2-cyclopenten-1-one	NIST	
1,2-Cyclopentanedione	NIST	
Methyl-1,2-cyclopentanedione	NIST	
1,2-cyclohexanedione	NIST	
2-hydroxy-2-cyclopenten-1-one	NIST	
Methyl-2-hydroxy-2-cyclopenten-1-one	NIST	
Methyl-2-cyclopenten-1-one	NIST	
Ethyl-2-hydroxy-2-cyclopenten-1-one	NIST	
Methyl-2-cyclopenten-1-one	NIST	
Dimethyl-2-cyclopenten-1-one	NIST	
4-cyclopentene-1,3-dione	NIST	
Methyl-2-Hexanone	NIST	
Furan	NIST	(alkyl)furan/pyran
3-furaldehyde	NIST	
2-furaldehyde	NIST	
2-acetyl-furan	NIST	
Methyl-2-furaldehyde	NIST	
(2H)-Furan3-one	NIST	
5-acetyldihydro-furanone	NIST	
Dihydro--furanone	NIST	
Methyl-furanone	NIST	
2(5H)-furanone	NIST	
2,5-Dimethyl-4-hydroxy-3(2H)-furanone (furaneol)	NIST	
4- Hydroxy-3-methyl-(5H)-furanone	NIST	
Methyl-2,5-Furandione,	NIST	
Dihydro- benzofuran	NIST	
3-Hydroxydihydro-2(3H)-furanone	NIST	
Dihydro-6-methyl-Pyranone	NIST	
Dihydro-3,5-dihydroxy-6-methyl-Pyranone	NIST	
3-hydroxy-2-methyl-(4H)-Pyran-4-on (Maltol)	NIST	
4-hydroxy-5,6-dihydro(2H)-pyran-2-one	[1]	
Methyl-benzofuran	NIST	
Furanmethanol	NIST	levosugars, anhydrosugars
Dianhydrorhamnose	[2]	
Anhydropentose/anhydro-cyclofuranose	[1]	
Anhydrohexose	[1]	
Levosugars(Levomannosan)	[1]	
Levosugars (Levoglucosan)	[1]	
Methyl-glucopyranoside, methyl	[1]	
**N compounds**		
Pyridine	NIST	(alkyl)pyridines/pyrroles
Pyrrole	NIST	
Methyl-pyrrole	NIST	
Tetrahydropyrrole	NIST	
1-methyl-pyrrole	NIST	
Trimethyl-pyrrole	NIST	
2-methyl-pyrrole	NIST	
3-ethyl-2,4-dimethyl-pyrrole	NIST	
3-methyl-pyrrole	NIST	
3-aminopyridine	NIST	

2-methylpyrimidine	NIST	
6-methyl-4(1H)-Pyrimidinone	NIST	
Diketodipyrrole	[4]	
Indole	NIST	
Methyl-indole	NIST	
Dimethyl-indole	NIST	
3-methyl-indole	NIST	
4-methyl-indole	NIST	
1,3-dihydro-indol-2-one	NIST	Aromatic N+ alkanenitriles+ alkylamides
9H-pyrido[3,4-b]indole	NIST	
Methyl-9H-pyrido[3,4-b]indole	NIST	
Benzylnitrile	NIST	
Benzeneacetonitrile	NIST	
Benzenepropanenitrile	NIST	
Alkanenitrile	NIST	
Alkylamide	NIST	
Hexadecanamide	NIST	
DKP Leu-Pro	[5, 6]	
DKP Phe-Pro	[5, 6]	
DKP Pro-Ala	[5, 6]	
DKP Pro-Gly	[5, 6]	
DKP Pro-Lys-NH3	[5, 6]	
DKP Pro-Met	[5, 6]	Proteins
DKP Pro-Phe	[5, 6]	
DKP Pro-Pro	[5, 6]	
DKP Pro-Val	[5, 6]	
2,6-Piperdinedione	NIST	
methyl 5-oxo-DL-prolinate	NIST	
**Alkanoic acids**		
Propanoic acid	NIST	
2-methyl-butanoic acid	NIST	
3-methyl-butanoic acid	NIST	
Butanoic acid	NIST	
Pentanoic acid	NIST	
Octanoic acid	NIST	
Nonanoic acid	NIST	
Nonanoic acid	NIST	
Dodecanoic acid	NIST	
Tetradecanoic acid	NIST	
Tetradecanoic acid methyl ester	NIST	
Tetradecanoic acid methyl-ethyl ester	NIST	
Pentadecenoic acid	NIST	
Pentadecanoic acid	NIST	
Hexadecenoic acid	NIST	Alkanoic acids
Hexadecanoic acid	NIST	
Hexadecanoic acid	NIST	
Hexadecanoic acid methyl ester	NIST	
Hexanediolic acid, bis (2-ethylhexyl) ester	NIST	
Hexadecanoic acid	NIST	
Heptadecenoic acid	NIST	
Heptadecanoic acid	NIST	
Octadecenoic acid	NIST	
Octadecatrienoic acid	NIST	
Octadecadienoic acid	NIST	
9-Octadecenoic acid (Z)-	NIST	
9-Octadecenoic acid, (E)-	NIST	
Octadecanoic acid	NIST	
Octadecanoic acid methyl ester	NIST	

trans-13-Octadecenoic acid	NIST	
Nonadecenoic acid	NIST	
Nonadecanoic acid	NIST	
Tetracosanoic acid methyl ester	NIST	
Tetracosanoic acid methyl ester	NIST	
Hexacosanoic acid methyl ester	NIST	
Octacosanoic acid methyl ester	NIST	
Triacontanoic acid methyl ester	NIST	
***n*-alkenes/*n*-alkanes**		
C9:1	NIST	
C11:1	NIST	
C13:1	NIST	
C14:1	NIST	
C15:1	NIST	
C16:1	NIST	
C17:1	NIST	
C18:1	NIST	
C19:1	NIST	
C20:1	NIST	
C21:1	NIST	*n*-alkenes
C22:1	NIST	
C23:1	NIST	
C24:1	NIST	
C25:1	NIST	
C26:1	NIST	
C27:1	NIST	
C29:1	NIST	
C30:1	NIST	
C31:1	NIST	
C33:1	NIST	
C13:0	NIST	
C14:0	NIST	
C15:0	NIST	
C16:0	NIST	
C17:0	NIST	
C18:0	NIST	
C19:0	NIST	
C20:0	NIST	
C21:0	NIST	
C22:0	NIST	
C23:0	NIST	*n*-alkanes
C24:0	NIST	
C25:0	NIST	
C26:0	NIST	
C27:0	NIST	
C28:0	NIST	
C29:0	NIST	
C30:0	NIST	
C31:0	NIST	
C32:0	NIST	
C33:0	NIST	
C35:0	*	
**Alkan-2-ones**		
Alkanone C13	NIST	
Alkanone C15	NIST	
Alkanone C16	NIST	Alkanones
Alkanone C17	NIST	
Alkanone C18	NIST	

Alkanone C19	NIST	
Alkanone C20	NIST	
Alkanone C21	NIST	
Alkanone C23	NIST	
Alkanone C25	NIST	
Alkanone C27	*	
Alkanone C28	*	
Alkanone C29	*	
Alkanone C31	*	
2-Pentadecanone, 6,10,14-trimethyl-	*	
Dibenzyl ketone	*	
**Phenols**		
Phenol	NIST	Phenols
2- methyl-phenol	NIST	
3- methyl-phenol	NIST	
4- methyl-phenol	NIST	
Dimethyl-phenol	NIST	
Dimethyl-phenol	NIST	
Ethyl- phenol	NIST	
3-ethyl- phenol	NIST	
4-ethyl- phenol	NIST	
2-propyl- phenol	NIST	
3-ethyl-5-methyl-phenol	NIST	
4-ethyl-2-methyl-phenol	NIST	
**Lignins**		
Guaiacol	NIST	Lignins
Acetoguaiacol	NIST	
Methyl-guaiacol	NIST	
Ethyl-guaiacol	NIST	
4-vinyl-guaiacol	NIST	
Propenyl-guaiacol	NIST	
Guaiacylacetone	NIST	
Hydroxy-propenyl-guaiacol	NIST	
Syringol	NIST	
Syringyl-acetone	NIST	
4-acetyl-syringol	NIST	
Acetosyringone	NIST	
Allenesyringol	NIST	
Propenyl-syringol	NIST	
4-formyl-syringol	NIST	
Propriosyringone	NIST	
Coniferyl alcohol	NIST	
Coniferol	NIST	
Dihydroconiferyl alcohol	NIST	
Benzeneacetic acid	NIST	
Benzenepropanoic acid	NIST	
Methyl ferulate	NIST	
Sinapic aldehyde	NIST	
Synapyl alcohol	NIST	
Methyl-ester-vanillic acid	NIST	
Vanillin	NIST	
Methyl ferulate	NIST	
Conipheryl alcohol	NIST	
**Chlorophylls**		
Prist-1-ene	[8]	Pristine, phytene, phytadiene
Phytadiene 1	[8]	
Phytadiene 2	[8]	
Phytol (3,7,11,15-Tetramethyl-2-hexadecen-1-ol)	NIST	

Phytol acetate	NIST	
Phytene	[8]	
Phytene	[8]	
**Steroids**		
Cholesterol	NIST	Steroids
5α-Cholestan-3β-ol	NIST	
Cholest-2-ene	NIST	
Cholest-3-ene	NIST	
Methyl-cholest-4-en-3-ol	NIST	
Cholest-4-en-3-one	NIST	
Cholest-4-ene	NIST	
Tetradecanoate-3β -cholest-5-en-3-ol	NIST	
3α-cholest-5-en-3-ol	NIST	
3β-cholest-5-en-3-ol	NIST	
Cholesta-3,5-diene	NIST	
3β-cholesta-4,6-dien-3-ol	NIST	
3α, 5β-cholestan-3-ol	NIST	
3β, 5β-cholestan-3-ol	NIST	
5β-cholestan-3-one	NIST	
Campesterol	NIST	
Ergostanol	NIST	
3β-ergost-5-en-3-ol	NIST	
5α-Ergost-8(14)-ene	NIST	
Sitosterol	NIST	
Stigmastanol	NIST	
Stigmasterol	NIST	
Stigmast-4-en-3-one	NIST	
Stigmastan-3,5-diene	NIST	
**Tocopherols**		
α-Tocopherol	NIST	Tocopherols
α-Tocopherol acetate	NIST	
β-Tocopherol	NIST	
γ-Tocopherol	NIST	
**Terpenoids**		
α-Amyrin	NIST	Terpenoids
β-Amyrin	NIST	
Limonene	NIST	
Squalene	NIST	
**(Poly)aromatics**		
Toluene	NIST	(Poly)aromatics
Styrene	NIST	
3,4-dimethoxytoluene	NIST	
1,4-methylbenzene	NIST	
2,3-dihydro-inden-1-one	NIST	
1,3-dihydro-inden-2-one	NIST	
Dimethyl-benzene	NIST	
Benzaldehyde	NIST	
4-hydroxy-3,5-dimethoxy-benzaldehyde	NIST	
Acetophenone	NIST	
1,4-Benzenediol	NIST	
1,2,4-Trimethoxybenzene	NIST	
1,2:4,5-Dibenzopyrene	NIST	

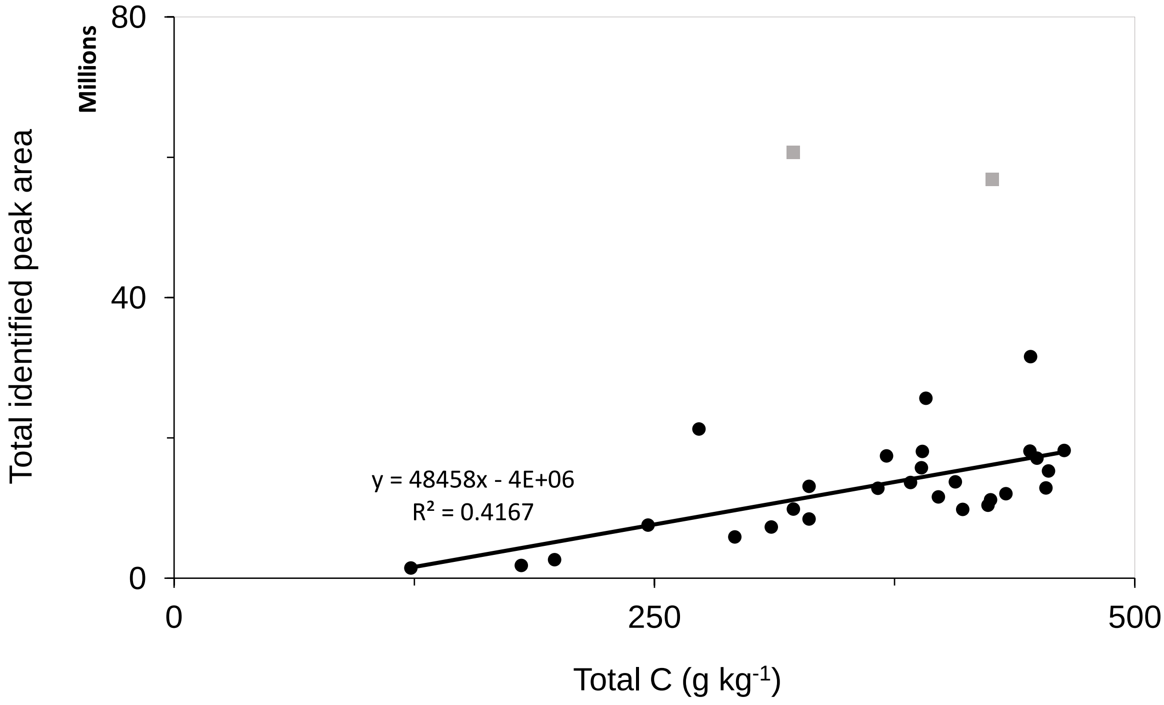

Figure S1. Correlation between total carbon (C) concentrations and total identified peak areas from the Py-GC/MS analysis of organic inputs. The total peak area has been normalized for the analyzed sample mass and corrected considering the split ratio (Table S2). Black circles indicate all samples included in the regression. Grey squares indicate the two samples considered as outliers and excluded from the regression. These were two process replicates of the same cattle slurry sample, i.e., “cattle slurry 2.” This sample had large peak area due mainly to the presence of *n*-alkanes and *n*-alkenes. The trend line for all samples included in the regression is shown with the interpolation equation and squared correlation coefficient (R^2 = 0.42).

Method S2. Digestion of organic inputs (solid and water-soluble pool)

Each organic input and solid certified reference material (CRM) was weighed (100-200 mg, 20 mg for cattle slurry samples) and digested in single replicates with 2 mL of HNO_3 (65%, double-distilled from EMSURE grade) and 2 mL of ultrapure water (> 18 MΩ cm, Nanopure Diamond™ system). For the water extracts, ~2 mL of sample was digested with 2 mL of HNO_3. The microwave digestion program was as follows: temperature ramp from 25 to 220 ˚C over 23 min (125 bar of pressure applied) followed by 8 min at 220 ˚C (125 bar of pressure applied). Prior to digestion, the 15 mL borosilicate glass digestion tubes were soaked for at least 24 hours in a warm bath (60 ˚C) containing 10% HNO_3 and rinsed thoroughly. Each digestion run included at least one procedural blank. After digestion, the digests were diluted with ultrapure water to a total volume of 10 mL, then filtered with 0.45 µm syringe filter (Perfect-Flow®, Nylon membrane), and stored at 4 °C until elemental quantification.

Method S3. Quantification of total elemental concentrations by ICP-MS(/MS)

Fe and Co in solid samples were quantified using an Agilent 7500 ICP-MS. The instrument included a He-supplied octopole reaction cell, a concentric nebulizer, a Scott double-pass spray chamber cooled to 2°C, and nickel sampler and skimmer cones. Fe (*m/z* 56), Co (*m/z* 59), and the internal standard containing Sc (*m/z* 45) were measured with 5 mL min^{-1} He in the reaction chamber, and acquisition parameters were 0.1 s integration time and 3 replicates.

All other elements, i.e., Na, Mg, K, Ca, P, Mn, Ni, Cu, Zn, Cd, and Ba, as well as Fe and Co in water extracts were quantified using an Agilent 8900 ICP-MS/MS. The instrument included a concentric nebulizer, a Scott double-pass spray chamber (2 °C), a high-throughput injection system (ISIS) with a PTFE sample loop, and nickel sampler and skimmer cones. To tune the ICP-MS/MS, a solution was used containing 10 µg L^{-1} of L, Y, Co, Ce, and Te (prepared from standards from J.T. Baker). To measure Na, Mg, K, Ca, Mn, Fe, Co, Ni, Cu, Zn, and Ba, single-quadrupole mode was used with 4.5 mL min^{-1} He. Cd (*m/z* 111->111) was measured with 5 mL min^{-1} H_2. P (*m/z* 31 ->47) and S (*m/z* 32->48) were measured in MS/MS mode with 1 mL min^{-1} H_2 and 30% O_2. Integration times were 0.05-0.3 ms (depending on element) and three replicates were collected per measured element. Quantification was performed by external calibration using standards from Bernd Kraft (Multi-element-standard 21 elements, multi-anion-standard 5 elements) and Merck 8 centripur. The calibration standards were prepared in 1% HNO_3 (i.e., the matrix of the analyzed samples after dilution). An internal standard containing Sc (70 µg L^{-1}), In (7 µg L^{-1}) and Lu (7 µg L^{-1}) was used to check signal stability during the measurements.

Table S4. Measured and certified elemental concentrations in solid samples. Analysis performed to validate ICP-MS(/MS) quantification of total elemental concentrations. The digested and analyzed solid CRM used for validation was WEPAL 232 (eucalyptus leaves). Average values of concentrations and recoveries are presented with error of ±1 standard deviation, as calculated from experimental (digested) replicates (ICP-MS Agilent 7500 n=3, ICP-MS/MS Agilent 8900 n=4).

Element	Instrument	Collision gas	Isotope	WEPAL 232 (Eucalyptus leaves)		
				$[element]_{measured}$ mg kg^{-1} (av ± sd)	$[element]_{certified}$ mg kg^{-1} (av ± sd)	Recovery % (av ± sd)
Na	Agilent 8900	He	23	3'030 ± 470	2965 ± 326	103 ± 16
Mg	Agilent 8900	He	24	1'765 ± 217	1660 ± 104	106 ± 13
K	Agilent 8900	He	39	6'025 ± 784	6120 ± 460	99 ± 13
Ca	Agilent 8900	He	44	13'484 ± 1'420	13100 ± 988	103 ± 11
P	Agilent 8900	O2	31 -> 47	1'038 ± 199	953 ± 66	109 ± 21
Mn	Agilent 8900	He	55	325.4 ± 41.5	324 ± 29.8	101 ± 13
Fe	Agilent 7500	He	56	76.9 ± 3.7	70.6 ± 8.59	109 ± 4
Co	Agilent 7500	He	59	0.0696 ± 0.0045	0.0621 ± 0.0167	110 ± 7
Ni	Agilent 8900	He	60	2.52 ± 0.45	2.51 ± 0.24	100 ± 18
Cu	Agilent 8900	He	63	6.9 ± 0.6	6.3 ± 0.783	110 ± 9
Zn	Agilent 8900	He	66	17.3 ± 2.6	14.7 ± 1.61	117 ± 18
Cd	Agilent 8900	H2	111 -> 111	0.0139 ± 0.0026	0.0147 ± 0.00431	90 ± 17
Ba	Agilent 8900	He	137	13.2 ± 2.1	13.9 ± 0.48	95 ± 15

Table S5. Measured and certified elemental concentrations in liquid samples. Analysis performed to validate ICP-MS/MS quantification of total elemental concentrations of water extracts. The liquid CRM NIST 1643f (freshwater) is presented. Average values of concentrations and recoveries are presented with error of ±1 standard deviation, as calculated from analytical replicates (n=3).

Element	Instrument	Collision gas	Isotope	NIST 1643f $[\text{element}]_{measured}$ μg L^{-1} (av ± sd)	$[\text{element}]_{certified}$ μg L^{-1} (av ± sd)	Recovery % (av ± sd)
Na	Agilent 8900	He	23	19265 ± 221	18830 ± 250	103 ± 1
Mg	Agilent 8900	He	24	8756 ± 89	7454 ± 60	119 ± 1
K	Agilent 8900	He	39	2205 ± 22	1933 ± 9	115 ± 1
Ca	Agilent 8900	He	44	33032 ± 253	29430 ± 330	113 ± 1
Mn	Agilent 8900	He	55	39.3 ± 0.3	37.14 ± 0.6	107 ± 1
Fe	Agilent 8900	He	56	109 ± 2	93.44 ± 0.78	118 ± 2
Co	Agilent 8900	He	59	26.4 ± 0.2	25.3 ± 0.17	105 ± 1
Ni	Agilent 8900	He	60	61 ± 1	59.8 ± 1.4	103 ± 2
Cu	Agilent 8900	He	63	25.3 ± 0.4	21.66 ± 0.71	118 ± 2
Zn	Agilent 8900	He	66	80 ± 1	74.4 ± 1.7	109 ± 2
Cd	Agilent 8900	H2	111 -> 111	6.14 ± 0.01	5.89 ± 0.13	105 ± 0
Ba	Agilent 8900	He	137	517 ± 5	518.2 ± 7.3	101 ± 1

Table S6. Solid-liquid (S:L) ratios used for water extractions of the organic inputs. Extraction was performed using ultrapure water (> 18 MΩ cm, Nanopure Diamond™ system). For each organic input type, the mean and ±1 standard deviation (SD) of all S:L ratios is provided. "Lignified crop residues" is abbreviated "LCR". "Farmyard manure" is abbreviated "FYM". "Industrial compost" here refers to "compost" in main manuscript.

Input type	Sample name	Sample mass (g)	Extractant volume (mL)	S:L	Mean ± 1SD
Green manure	Berseem clover	0.13566	10	74	97 ± 26
	Black oat	0.11555	10	87	
	Phacelia	0.12239	10	82	
	Subterranean clover	0.06692	10	149	
	Yellow mustard	0.12212	10	82	
	Green manure mix 1	0.08774	10	114	
	Green manure mix 2	0.11101	10	90	
LCR/Litter	Barley straw	0.10995	10	91	84 ± 17
	Shredded corn	0.17095	10	58	
	Wheat straw	0.10326	10	97	
	Poultry litter	0.11342	10	88	
Monogastric FYM	Pig manure (fresh)	0.17238	10	58	62 ± 4
	Pig manure (pile)	0.16349	10	61	
	Pig slurry	0.1457	10	69	
	Poultry manure	0.16168	10	62	
Ruminant FYM	Cattle manure	0.22742	10	44	47 ± 4
	Horse manure	0.20328	10	49	
	Mixed manure pile	0.2244	10	45	
	Composted cattle manure 1	0.22063	10	45	
	Composted cattle manure 2	0.20746	10	48	
	Composted catte manure 3	0.18661	10	54	
Cattle slurry	Cattle slurry 1	0.04063	10	246	194 ± 50
	Cattle slurry 2	0.05335	10	187	
	Cattle slurry 3	0.06775	10	148	
Industrial compost	Gerber compost 1	0.2258	10	44	19 ± 17
	Gerber compost 2	0.72124	10	14	
	Gerber compost 3	1.17257	10	9	
	Biomassehof compost	1.17076	10	9	

Method S4. SEC-UV-ICP-MS/MS analysis of organic input water extracts

To perform size exclusion chromatography coupled to ultraviolet detection and triple-quadrupole inductively coupled plasma mass spectrometry (SEC-UV-ICP-MS/MS), an Agilent 1260 Infinity II high performance liquid chromatography (HPLC) system was coupled to an Agilent diode array detector (UV, 254 nm) and an Agilent 8900 ICP-MS/MS. ICP-MS/MS operating conditions were as described in Method S3. The SEC columns included one Shodex OH-pak SB-803 column and one SB-802.5 HQ column (separation <100 kDa and ~40 nm). The mobile phase used for SEC separation was ammonium nitrate (5 mmol L^{-1}, pH 8, flow rate of 1 mL min^{-1}, injection volume of 100 µL).

Each organic input extract was measured by SEC-UV-ICP-MS/MS twice using different gases in the collision/reaction cell to obtain speciation data on Zn and Cd together with various elements. Firstly, 5 mL min^{-1} He gas was used to detect Zn (*m/z* 66; 50 ms integration time) and Fe (*m/z* 56->56; 25 ms). Secondly, 25% O_2 and 1 mL min^{-1} H_2 was used to detect C (*m/z* 12 ->28; 50 ms), P (*m/z* 31->47, 25 ms), S (*m/z* 32->48; 50 ms), and Cd (*m/z* 111->111, 50 ms). For each run, an internal standard containing scandium (Sc; 450 µg L^{-1}), indium (In; 45 µg L^{-1}) and lutetium (Lu; 45 µg L^{-1}) was injected with the sample into the ICP-MS/MS to monitor any changes in instrument sensitivity. After SEC-UV-ICP-MS/MS measurements, peak deconvolution of each chromatogram (Zn, Cd, P, S, Fe, and UV) from each organic input extract was performed using Origin 2021 (Fit peak pro), as outlined by Laborda et al.

Part II: Supplement to results and discussion

Table S7. Output of the hierarchical cluster analysis performed on bulk properties. Analysis was performed using ward linkages in SPSS Statistics23 from IBM. Solutions for selection of four, five, and six clusters are presented. The division lines in the table highlight the cluster number presented in the main text (i.e., 4 clusters). Parameters used for cluster analysis included pH and total C, N, S, Zn, and Cd contents. "Lignified crop residues" is abbreviated "LCR". "Farmyard manure" is abbreviated "FYM". "Industrial compost" here refers to "compost" in main manuscript.

Sample type	Sample name	Members (6 clusters)	Members (5 clusters)	Members (4 clusters)
Green manure	Berseem clover	1	1	1
Green manure	Black oat	2	1	1
Green manure	Phacelia	2	1	1
Green manure	Subterranean clover	2	1	1
Green manure	Green manure mix 1	2	1	1
Green manure	Green manure mix 2	2	1	1
Green manure	Yellow mustard	2	1	1
Ruminant FYM	Horse manure	2	1	1
Industrial compost	Gerber compost 1	2	1	1
LCR/Litter	Poultry litter	2	1	1
LCR/Litter	Barley straw	3	2	2
LCR/Litter	Shredded corn	3	2	2
LCR/Litter	Wheat straw	3	2	2
Monogastric FYM	Poultry manure	4	3	3
Ruminant FYM	Cattle manure	4	3	3
Ruminant FYM	Mixed manure pile	4	3	3
Ruminant FYM	Composted cattle manure 1	4	3	3
Ruminant FYM	Composted cattle manure 2	4	3	3
Ruminant FYM	Composted cattle manure 3	4	3	3
Cattle slurry	Cattle slurry 1	4	3	3
Cattle slurry	Cattle slurry 2	4	3	3
Cattle slurry	Cattle slurry 3	4	3	3
Monogastric FYM	Pig manure (fresh)	5	4	3
Monogastric FYM	Pig manure (pile)	5	4	3
Monogastric FYM	Pig slurry	5	4	3
Industrial compost	Gerber compost 2	6	5	4
Industrial compost	Gerber compost 3	6	5	4
Industrial compost	Biomassehof compost	6	5	4

Table S8. Summary of literature values for the total concentrations of Zn and Cd in organic inputs.[4–12] The number of each organic input type sampled in each study (n) is provided, in addition to the citation reference and the calculated Zn-to-Cd (Zn:Cd) mass ratios. Some values were found to be below the limits of the detection (<LOD) of the analytical technique employed by the study. The organic input type "lignified crop residues and litter" is abbreviated as "LCR/Litter," while "farmyard manure" is abbreviated as "FYM". "Industrial compost" here refers to "compost" in main manuscript.

Input type	Sample name(s)	n	Citation	Total Zn (mg/kg)	Total Cd (mg/kg)	Zn:Cd mass ratio
Green manure	White mustard, berseem clover (grown in soils with varying Zn bioavailability)	6	Grueter et al. (2018)	8 - 119	0.1 - 0.8	11 - 572
LCR/Litter	Sawdust	1	Meng et al. (2017)	10.0 ± 0.6		
LCR/Litter	Corn stalk	1	Yan et al. (2022)	8.0 ± 0.6	0.14 ± 0.01	57
Monogastric FYM	Poultry manure from University of Agriculture Faisalabad, Pakistan	1	Azhar et al. (2019)	129	1.3	99
Monogastric FYM	Pig manures	305	Hoelzel et al. (2012)	93 - 8239	0.1 - 5.3	
Ruminant FYM	Cattle manures	48	Zhang et al. (2012)	17 - 377	<LOD - 10	
Ruminant (cattle) slurry	Dairy cattle slurry from Pavia, Italy	1	Provolo et al. (2018)	204 ± 23		
Ruminant (cattle) slurry	Cattle slurry from CRUCIAL field trial, Denmark	1	Poulsen et al. (2013)	229 ± 45	0.2 ± 0.0	1145
Industrial compost	Input materials included green waste, sewage sludge, and woody waste	4	Greenway and Song (2002)	26 - 355	0.2 - 3.2	111 - 199

Table S9. Biochemical class and organic matter (OM) molecular composition summary for six clusters based on the Py-GC/MS dataset. Data is presented as relative abundance of total identified pyrolytic products (%). The freshness index of carbohydrate is presented in percentage (%) and was calculated as 100 * (levosugars, anhydrosugars)/sum of degraded carbohydrates (cyclo-hex-pentanone, furans, pyrans). The freshness index of N compounds is presented in percentage (%) and was calculated as proteins 100 * (diketopiperazine)/sum of degraded N compounds (aromatic N compounds, alkylamides, alkanenitriles). For each organic input type, the mean ±1 standard deviation, minimum, and maximum values are provided. The organic input type “lignified crop residues and litter” is abbreviated as “LCR/Litter,” “monogastric” is abbreviated “mono”, and “farmyard manure” is abbreviated “FYM”. “Industrial compost” here refers to “compost” in main manuscript.

	**Cluster 1**	**Cluster 2**	**Cluster 3**	**Cluster 4**	**Cluster 5**	**Cluster 6**
	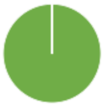	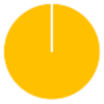	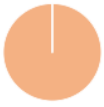	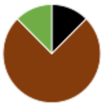	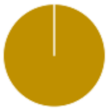	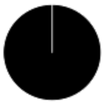
	n = 6	n = 4	n = 4	n = 8	n = 3	n = 3
**Carbohydrates**						
Levosugars, anhydrosugars	4 ± 6<br>*1 - 16*	6 ± 4<br>*2 - 10*	1.8 ± 0.4<br>*1.2 - 2.2*	1 ± 1<br>*0 - 4*	0 ± 0<br>*0 - 0*	2 ± 1<br>*2 - 3*
(alkyl)furan, (alkyl)pyran	11 ± 3<br>*6 - 15*	18 ± 4<br>*14 - 23*	5 ± 3<br>*3 - 9*	6 ± 4<br>*1 - 12*	1 ± 1<br>*0 - 2*	6.9 ± 1.1<br>*5.9 - 8.0*
Cyclohexanone, cyclopentanone	13 ± 2<br>*10 - 17*	15 ± 3<br>*11 - 16*	6 ± 4<br>*3 - 11*	8 ± 3<br>*5 - 14*	4 ± 2<br>*3 - 6*	5 ± 1<br>*4 - 7*
Freshness index	24 ± 38<br>*5 - 102*	20 ± 14<br>*6 - 37*	22 ± 12<br>*9 - 32*	9 ± 7<br>*2 - 20*	0 ± 0<br>*0 - 0*	17 ± 3<br>*16 - 20*
Total	29 ± 4<br>*25 - 34*	39 ± 7<br>*31 - 48*	12 ± 6<br>*8 - 21*	15 ± 8<br>*7 - 27*	5 ± 3<br>*3 - 8*	14 ± 3<br>*12 - 18*
**N compounds**						
Proteins (diketopiperazine)	4 ± 1<br>*2 - 5*	0.13 ± 0.05<br>*0.05 - 0.18*	1.0 ± 1.5<br>*0.1 - 3.2*	1.2 ± 0.8<br>*0.1 - 2.8*	0.97 ± 0.09<br>*0.86 - 1.03*	2.1 ± 0.4<br>*1.7 - 2.5*
(alkyl)pyridines/pyrroles	1.8 ± 0.6<br>*0.9 - 2.5*	0.7 ± 0.2<br>*0.5 - 1.0*	1 ± 1<br>*0 - 2*	2 ± 1<br>*1 - 4*	1.2 ± 0.5<br>*0.9 - 1.8*	5.8 ± 0.3<br>*5.4 - 6.0*
Aromatic N, alkylamids, and alkanenitriles	3.9 ± 1.0<br>*2.7 - 5.2*	0.6 ± 0.2<br>*0.4 - 0.8*	2 ± 2<br>*1 - 4*	3 ± 1<br>*1 - 5*	6.7 ± 0.5<br>*6.4 - 7.3*	5.0 ± 0.6<br>*4.4 - 5.6*
Freshness index	*62 ± 17*<br>*35 - 81*	*10 ± 4*<br>*6 - 14*	*31 ± 48*<br>*4 - 102*	*22 ± 13*<br>*3 - 38*	*12 ± 2*<br>*10 - 14*	*19 ± 3*<br>*16 - 22*
Total	9 ± 2<br>*7 - 12*	1.4 ± 0.4<br>*0.9 - 1.9*	4 ± 2<br>*1 - 6*	7 ± 2<br>*3 - 10*	9 ± 0<br>*8 - 9*	13 ± 1<br>*12 - 14*
**Chlorophyll**	7 ± 2<br>*3 - 9*	0.21 ± 0.14<br>*0.03 - 0.37*	0.9 ± 0.5<br>*0.4 - 1.4*	2.4 ± 1.2<br>*0.9 - 3.6*	4.9 ± 1.0<br>*3.8 - 5.6*	5 ± 1<br>*4 - 6*
**Lignins**	18 ± 6<br>*8 - 24*	50 ± 7<br>*40 - 56*	16 ± 8<br>*8 - 26*	55 ± 10<br>*41 - 69*	9 ± 7<br>*4 - 17*	39 ± 0<br>*39 - 39*
**Phenols**	5.8 ± 0.7<br>*4.8 - 6.4*	2.7 ± 0.8<br>*1.8 - 3.7*	2 ± 1<br>*1 - 3*	6 ± 2<br>*3 - 8*	8.9 ± 1.0<br>*8.0 - 9.9*	9 ± 1<br>*8 - 10*
**Alkanoic acids**	17 ± 4<br>*11 - 23*	2.0 ± 0.5<br>*1.5 - 2.6*	55 ± 20<br>*32 - 75*	4 ± 3<br>*1 - 8*	3 ± 1<br>*2 - 4*	4 ± 0<br>*3 - 4*
**Alkan-2-ones**	0.29 ± 0.05<br>*0.25 - 0.38*	0.29 ± 0.20<br>*0.01 - 0.48*	0.4 ± 0.8<br>*0.0 - 1.7*	0.2 ± 0.1<br>*0.0 - 0.4*	7.0 ± 3.6<br>*2.9 - 9.3*	0 ± 0<br>*0 - 0*
***n*-alkanes/*n*-alkenes**	5.6 ± 2.1<br>*3.5 - 9.6*	0.80 ± 0.78<br>*0.07 - 1.68*	1.9 ± 2.4<br>*0.6 - 5.5*	3 ± 2<br>*1 - 5*	24.3 ± 1.8<br>*23.2 - 26.4*	10 ± 2<br>*8 - 12*
**Steroids**	3.7 ± 0.9<br>*2.1 - 4.4*	1.5 ± 0.3<br>*1.2 - 1.9*	5.7 ± 1.1<br>*4.4 - 6.8*	1.5 ± 0.9<br>*0.5 - 2.7*	11 ± 0<br>*11 - 12*	0.3 ± 0.0<br>*0.3 - 0.4*
**(poly)aromatics**	4.1 ± 0.8<br>*3.0 - 5.3*	2.1 ± 0.7<br>*1.1 - 2.6*	1 ± 1<br>*0 - 3*	3.7 ± 1.7<br>*1.6 - 6.9*	10.1 ± 2.8<br>*6.9 - 12.0*	5 ± 1<br>*4 - 5*
**Terpenoids**	0.1 ± 0.2<br>*0.0 - 0.5*	0.1 ± 0.1<br>*0.0 - 0.3*	0.2 ± 0.1<br>*0.1 - 0.3*	1.7 ± 1.0<br>*0.3 - 3.0*	6 ± 1<br>*5 - 7*	1.3 ± 0.3<br>*1.1 - 1.6*
**Tocopherol**	0.5 ± 0.1<br>*0.3 - 0.7*	0.10 ± 0.05<br>*0.02 - 0.14*	0.6 ± 0.1<br>*0.4 - 0.8*	0.6 ± 0.2<br>*0.2 - 1.0*	1.0 ± 0.3<br>*0.42 - 0.76*	0.3 ± 0.1<br>*0.2 - 0.4*

■ Green manure ■ LCR/Litter ■ Mono FYM ■ Ruminant FYM ■ Cattle slurry ■ Industrial compost

Table S10. Output of the hierarchical cluster analysis performed with the Py-GC/MS dataset. Analysis was performed using ward linkages in SPSS Statistics23 from IBM. Solutions for selection of four, five, and six clusters are presented. The division lines in the table highlight the cluster number presented in the main text (i.e., 6 clusters). "Lignified crop residues" is abbreviated "LCR". "Farmyard manure" is abbreviated "FYM". "Industrial compost" here refers to "compost" in main manuscript.

Sample type	Sample name	Members (6 clusters)	Members (5 clusters)	Members (4 clusters)
Green manure	Berseem clover	1	1	1
Green manure	Black oat	1	1	1
Green manure	Phacelia	1	1	1
Green manure	Subterranean clover	1	1	1
Green manure	Green manure mix 1	1	1	1
Green manure	Green manure mix 2	1	1	1
LCR/Litter	Barley straw	2	2	2
LCR/Litter	Shredded corn	2	2	2
LCR/Litter	Wheat straw	2	2	2
LCR/Litter	Poultry litter	2	2	2
Monogastric FYM	Pig manure (fresh)	3	3	3
Monogastric FYM	Pig manure (pile)	3	3	3
Monogastric FYM	Pig slurry	3	3	3
Monogastric FYM	Poultry manure	3	3	3
Ruminant FYM	Cattle manure	4	4	2
Ruminant FYM	Horse manure	4	4	2
Ruminant FYM	Mixed manure pile	4	4	2
Ruminant FYM	Composted cattle manure 1	4	4	2
Ruminant FYM	Composted cattle manure 2	4	4	2
Ruminant FYM	Composted catte manure 3	4	4	2
Industrial compost	Gerber compost 1	4	4	2
Green manure	Yellow mustard	4	4	2
Cattle slurry	Cattle slurry 1	5	4	2
Cattle slurry	Cattle slurry 2	5	4	2
Cattle slurry	Cattle slurry 3	5	4	2
Industrial compost	Gerber compost 2	6	5	4
Industrial compost	Gerber compost 3	6	5	4
Industrial compost	Biomassehof compost	6	5	4

Table S11. Percentage of water-soluble elements (in % total element content) in the organic input clusters grouped according to the cluster analysis performed with the water-soluble Zn and Cd speciation dataset. Values include carbon (C), phosphorus (P), sulfur (S), iron (Fe), zinc (Zn), and cadmium (Cd) (expressed in % of total C, P, S, Fe, Zn, and Cd, respectively). Error is presented as ±1 standard deviation for “n” number of samples for each organic input type. Two samples, phacelia (green manure) and barley straw (LCR/Litter) contained water-soluble Fe values below the limit of the detection of our method (<LOD). “Industrial compost” here refers to “compost” in main manuscript.

**Water-extractable (%)**	**Cluster 1**	**Cluster 2**	**Cluster 3**	**Cluster 4**	**All (% RSD)**
	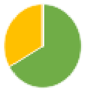	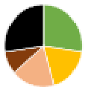	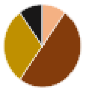	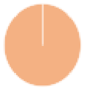	
	**n = 6**	**n = 11**	**n = 10**	**n = 1**	**n = 28**
C	22 ± 12	14 ± 12	14 ± 7	8	16 ± 10 (66)
	6 - 37	*3 - 40*	*1 - 24*		*1 - 40*
P	10 ± 4	7 ± 6	4 ± 2	3	6 ± 5 (80)
	4 - 15	*0.5 - 21.5*	*1 - 7*		*0.5 - 21.5*
S	16 ± 9	31 ± 60	14 ± 7	5	21 ± 38 (181)
	6 - 30	*2 - 209*	*4 - 32*		*2 - 209*
Fe	2 ± 3	3 ± 5	5 ± 3	0.9	4 ± 4 (111)
	<LOD - 7	*<LOD - 15*	*0.4 - 8.2*		*<LOD - 15*
Zn	52 ± 28	13 ± 18	6 ± 3	1.5	18 ± 25 (133)
	6 - 89	*0.7 - 50.0*	*0.7 - 10.4*		*0.7 - 89.2*
Cd	25 ± 28	11 ± 11	12 ± 7	0.4	14 ± 16 (112)
	3 - 79	*0.9 - 36.3*	*2 - 25*		*0.4 - 79.2*

▪ Green manure ▪ LCR/Litter ▪ Mono FYM ▪ Ruminant FYM ▪ Cattle slurry ▪ Industrial compost

Note S3: Separation of trace element compounds by size exclusion chromatography

The SEC conditions employed in this study (Shodex OH-Pak SB 803 & 802.5 columns with 5 mM ammonium nitrate) were thoroughly optimized previously, i.e., different SEC columns and mobile phase compositions were tested, to reach the best chromatographic resolution and recoveries of trace elements in soil extracts targeting the water-soluble and organic matter fractions.[13] In a previous study by Tolu et al.,[13] the series of Shodex OH-Pak SB 803 & 802.5 columns (targeting a size range between <1-100 kDa) was more optimal than the Superdex peptide 10/300 GL column (<1-7 kDa), with which many elements eluted in the dead volume and were not properly separated. The series of Shodex OH-Pak SB 803 & 802.5 columns was also more optimal than the Agilent PL-Aquagel-OH 30 (1-100 kDa), because it allowed better separation of late-eluting trace elements (i.e., molecules of lower molecular weight -LMW-). In addition, although increasing NH_4NO_3 concentration in the mobile phase above 5 mM (e.g., to 25 mM) reduced the secondary ionic interactions that impede pure size separation of analytes (i.e., avoiding that negatively charged compounds elute earlier due to ionic repulsion with negatively charged SEC column phase), a mobile phase composed of 5 mM ammonium nitrate (NH_4NO_3) was determined to be ideal. Indeed, mobile phase NH_4NO_3 concentrations above 5 mM led to a drastic decrease in both trace element recoveries and separation resolution due to OM aggregation with increasing mobile phase ionic strength. Finally, the authors of Tolu et al. tested addition of different amounts of methanol in the mobile phase to check for potential secondary hydrophobic interactions, another barrier to pure size separation of analytes. However, the results showed that increasing methanol (MeOH) concentrations did not have a perceivable effect on trace element separation, indicating no hydrophobic interaction of trace elements and organic matter with the optimal SEC columns (Shodex OH-Pak SB 803 & 802.5) in soil extracts. Because addition of MeOH to the mobile phase decreases the sensitivity of ICP-MS/MS detection (due to the requirement of adding Ar/O_2 gases mixture in the plasma), a mobile phase without MeOH was preferred.

Overall, with the optimal SEC conditions from Tolu et al. (used here for analysis of our organic input water extracts), ionic interactions between the analytes and the negatively charged stationary phase of the columns remain. Indeed, Figure S2a shows that the negatively-charged polystyrene sulfonate molecular weight standards (PSS) eluted earlier than the neutrally charged pullulan standards with the used SEC conditions due to repulsion effects of strongly negatively charged species. Also, free Zn^{2+} and Cd^{2+} did not elute out due to ionic retention. Achieving a separation of trace element compounds and nanoparticles solely based on size remains, however, a common challenge.[14,15] Despite the ionic interactions occurring during our SEC separation, isolated and purified humic acids eluted earlier with our optimized SEC than fulvic acids (Figure S2b,c), which is consistent with the higher average molecular weight of humic versus fulvic acids. Thus, dissolved organic matter (OM) is separated by size with the SEC method employed in this study. Moreover, Tolu et al. (2022) showed the early elution and separation from the OM fractions of mineral nanoparticles containing Fe, Mn, As, and Pb and of size >20-40 nm (based on column manufacturer specifications and comparison of SEC data with <20 nm filtration for soil extracts targeting the water-soluble and organic matter fractions).

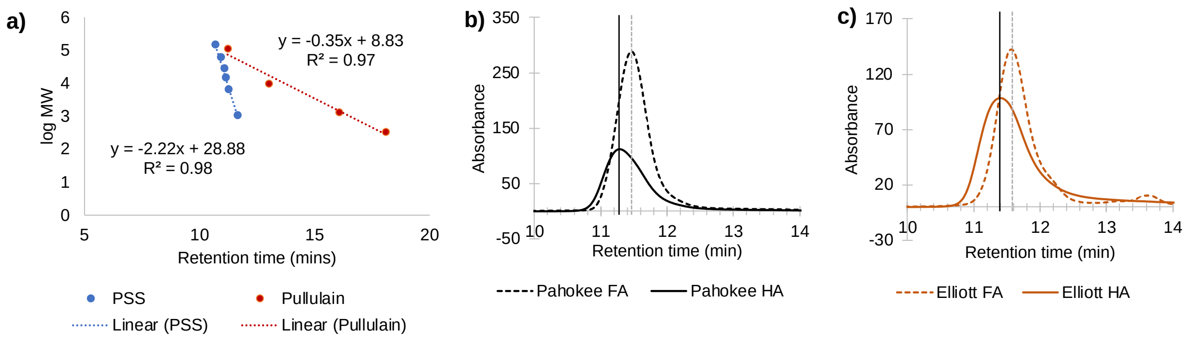

Figure S2. Molecular weight and humic standards analyzed with size exclusion chromatography coupled to UV and ICP-MS/MS. Analysis of these standards provides additional information on the SEC separation of the operating conditions employed in this study. Panel a) shows the size calibration obtained using molecular weight polystyrene sulfonate standards (negatively charged) and pullulan standards (neutral), with the log of the molecular weight (MW) in Daltons plotted against retention time. Panels b) and c) show the UV absorbance chromatograms obtained with the optimized SEC separation for isolated and purified humic and fulvic acid materials from the International Humic Substances Society (from Pahokee peat and Elliott soil, respectively).

Water-soluble pool of organic inputs

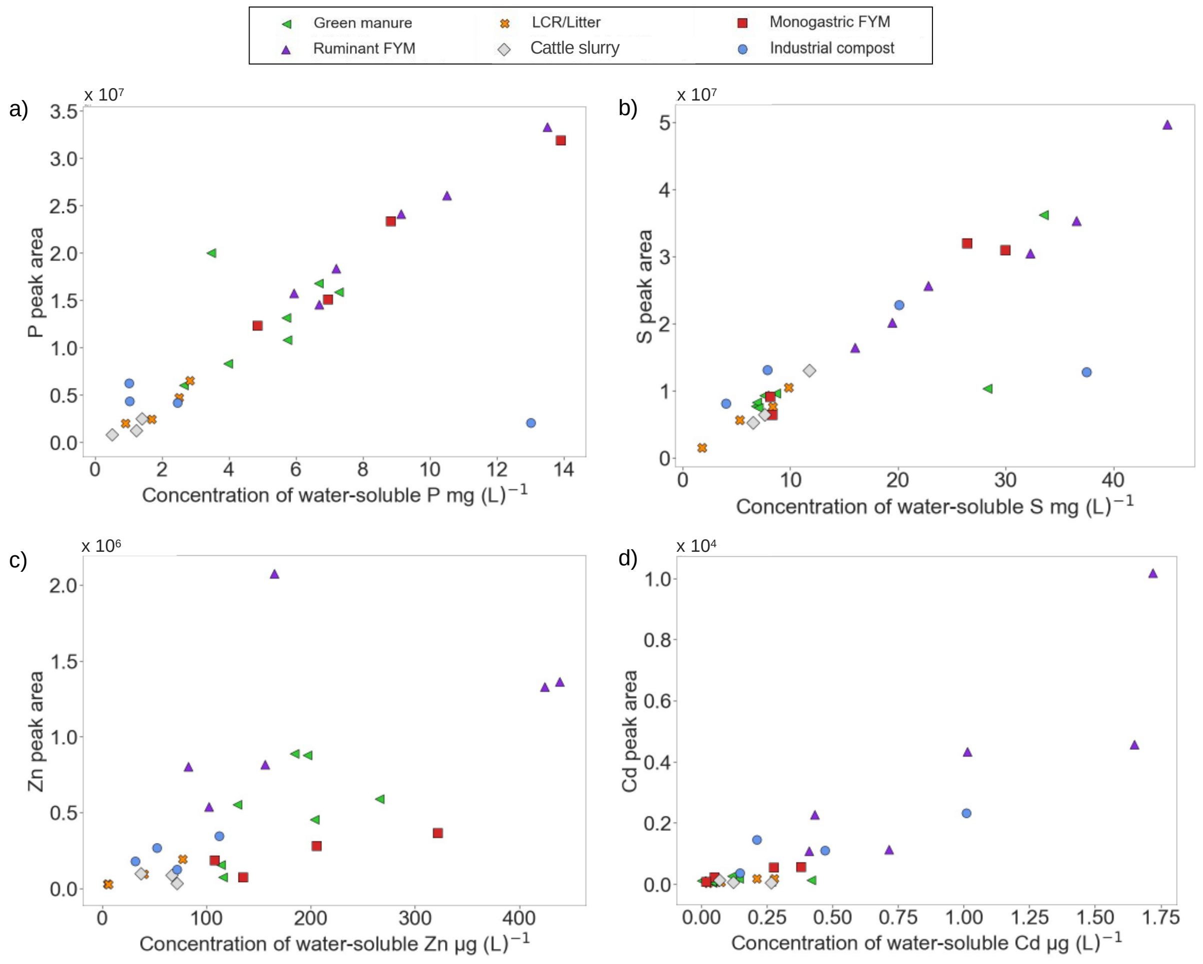

Figure S3. Correlations between the sum of element SEC peak area and total concentrations in the water extracts. Data is presented for phosphorus (P; panel a), sulfur (S; panel b), zinc (Zn; panel c), and cadmium (Cd; panel d). The shown correlations are statistically significant (2-tailed Spearman's bivariate correlation) with an r^2 and p-value of, respectively, 0.80 and <0.0001 for P, 0.86 and <0.0001 for S, 0.72 and <0.0001 for Zn, and 0.78 and <0.0001 for Cd. A total of n = 28 organic input samples were measured. The organic input type "lignified crop residues and litter" is abbreviated as "LCR/Litter," while "farmyard manure" is abbreviated "FYM". "Industrial compost" here refers to "compost" in main manuscript.

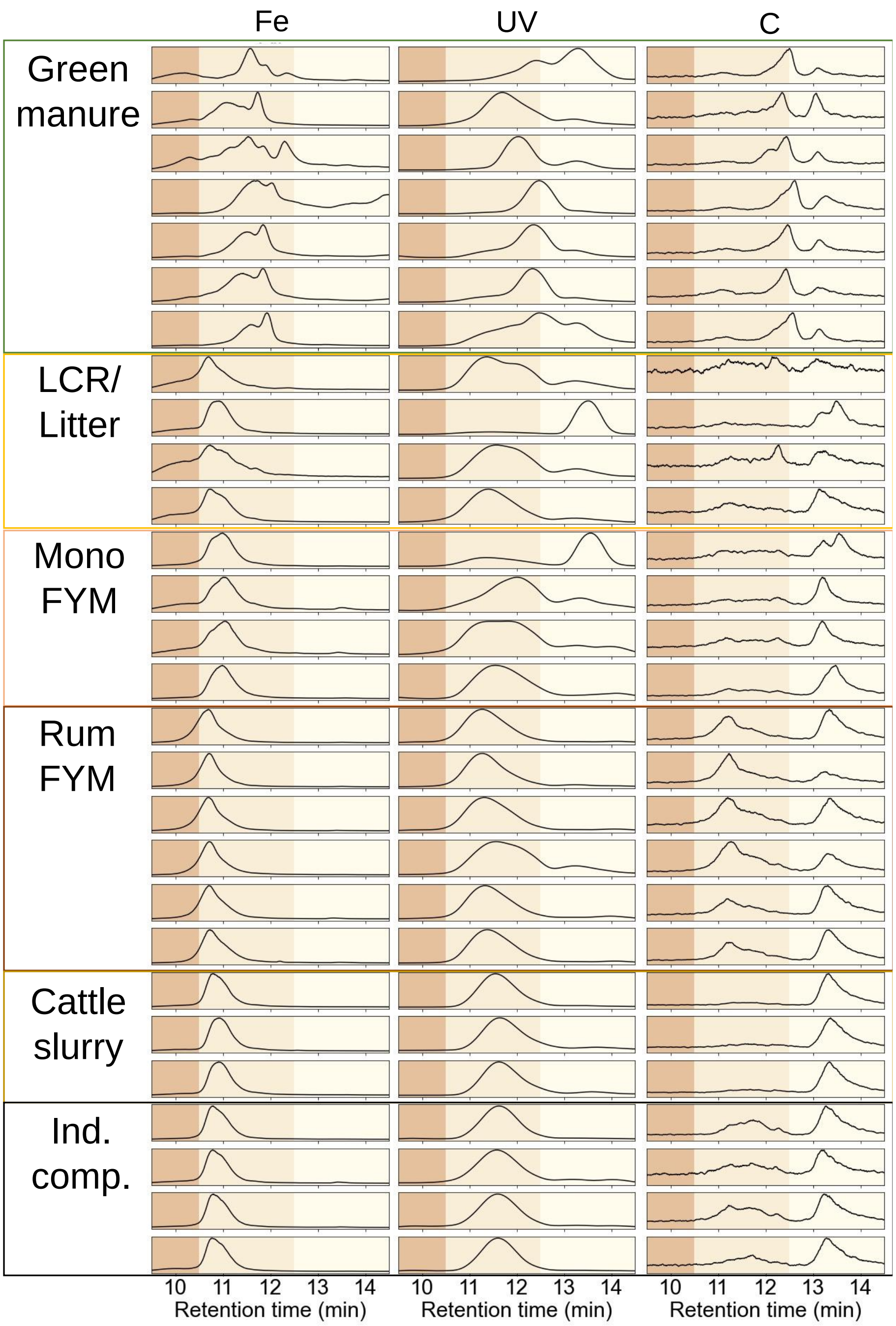

Figure S4. Chromatograms showing Fe, UV, and C elution and intensity in all organic input samples. Measurements were performed using SEC-UV-ICP-MS/MS. Data is presented with separation based on organic input types, including green manure, lignified crop residues and litter (LCR/Litter), monogastric farmyard manure (mono FYM), ruminant farmyard manure (rum FYM), ruminant cattle slurry (cattle slurry), and industrial compost (ind. comp., which refers to “compost” in main manuscript). Shaded regions indicate general time ranges for peak apexes in F1 (oxyhydroxide nanoparticles, 9.5-10.5 mins), F2 (higher-molecular-weight OM, 10.5-12.5 mins), and F3 (lower-molecular-weight OM, 12.5-14.5 mins).

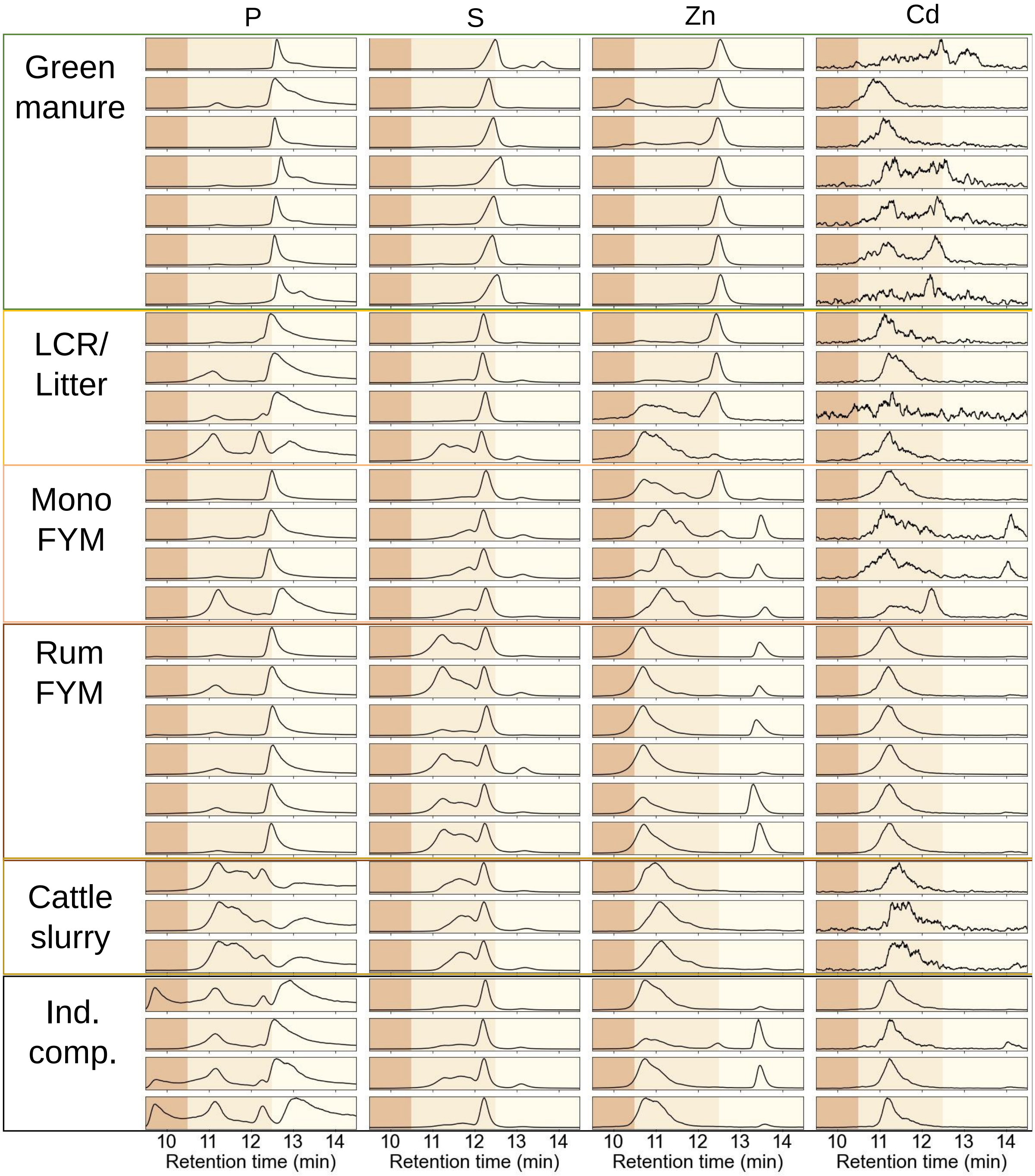

Figure S5. Chromatograms showing P, S, Zn, and Cd elution and intensity in all organic input samples. Measurements were performed using SEC-UV-ICP-MS/MS. Data is presented with separation based on organic input types, including green manure, lignified crop residues and litter (LCR/Litter), monogastric farmyard manure (mono FYM), ruminant farmyard manure (rum FYM), ruminant cattle slurry (cattle slurry), and industrial compost (ind. comp., which refers to “compost” in main manuscript). Shaded regions indicate general time ranges for peak apexes in F1 (oxyhydroxide nanoparticles, 9.5-10.5 mins), F2 (higher-molecular-weight OM, 10.5-12.5 mins), and F3 (lower-molecular-weight OM, 12.5-14.5 mins). Note that peaks of Zn and Cd eluting with peak apexes ~12.5 mins were considered as F3.

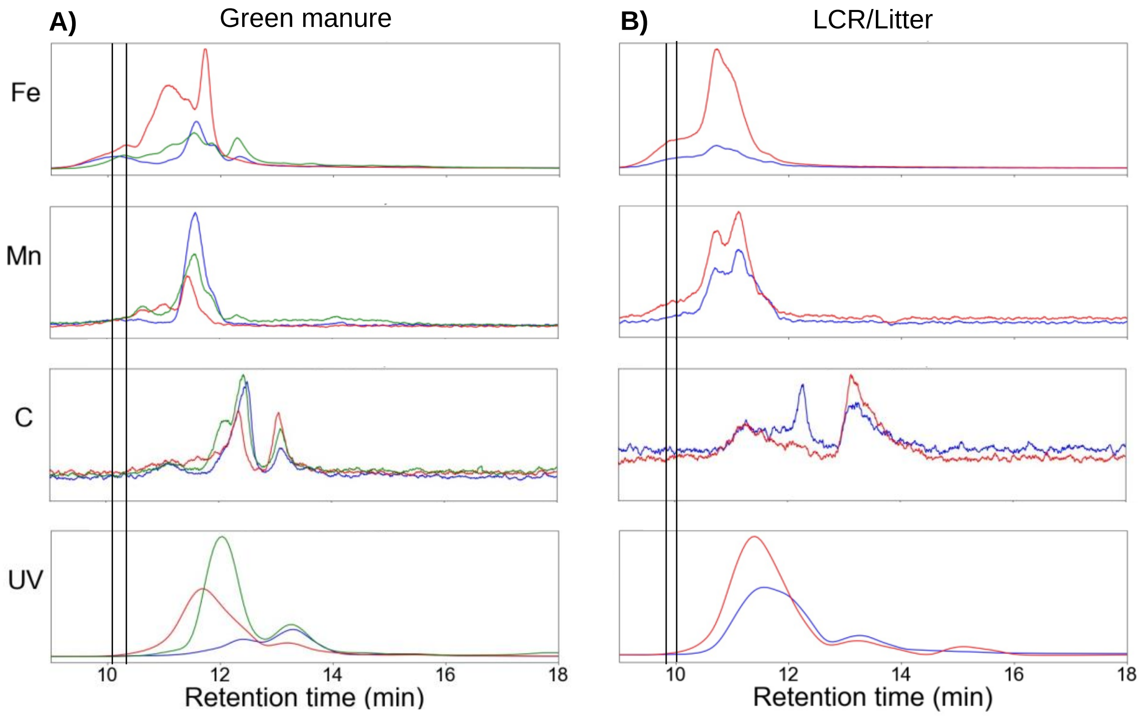

Figure S6. Definition of SEC fraction 1 (F1) in water extracts of organic inputs. Illustrated using chromatograms of Fe, Mn, C, and UV absorbance. This fraction was defined by co-elution of Fe and Mn (black vertical line) in the absence of peaks of C and UV. Evidence of the presence of F1 is shown by plotting three chromatograms of green manure samples (panel A; yellow mustard, subterranean clover, black oat) and two LCR/Litter samples (panel B; barley straw, shredded corn). F1 was identified as containing (oxy)hydroxides nanoparticles and was characterized by small peaks of Fe and Mn detected in the absence of both C or UV peaks. Peak apexes were found at retention times <10.5 mins.

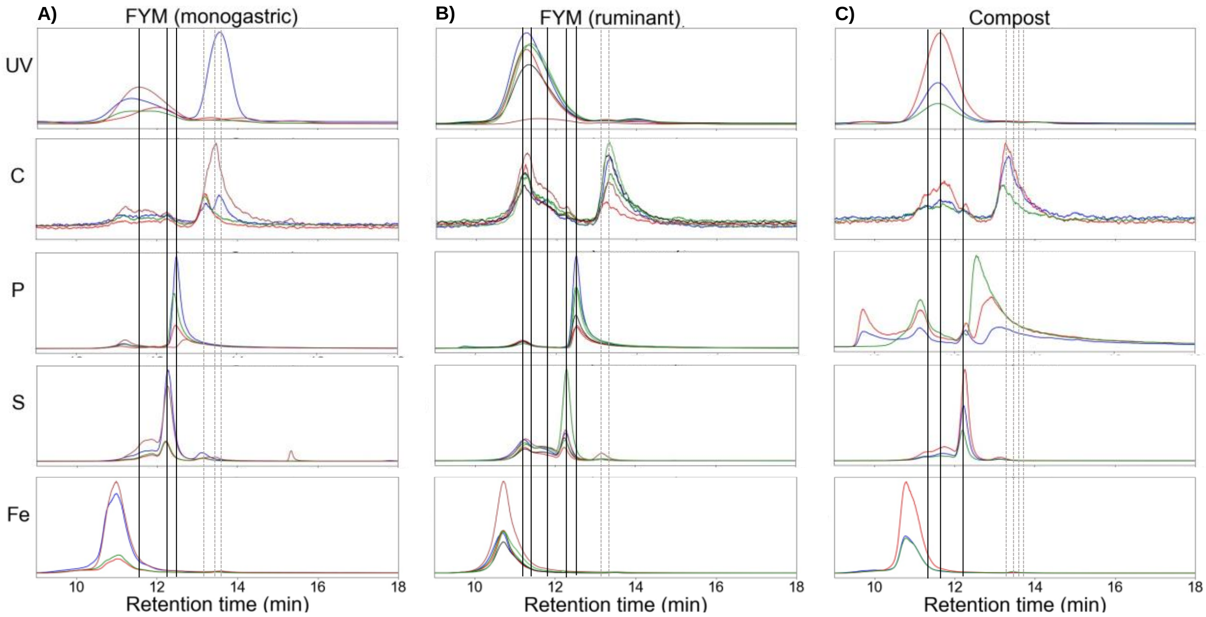

Figure S7. Definition of SEC fraction 2 (F2) and fraction 3 (F3) in water extracts of organic inputs. Illustrated using chromatograms of UV absorbance, C, and S. Fraction F2 was defined by co-elution of UV and C (gray vertical lines) as well as sharp peaks of S. F2 is shown by plotting all chromatograms of FYM (monogastric) samples (panel A), FYM (ruminant) samples (panel B), and compost samples (panel C). F3 was defined by strong C features (dashed vertical lines) as well as peaks of UV and P in select samples. F3 is shown by plotting chromatograms of FYM (monogastric) samples (panel A), FYM (ruminant) samples (panel B), and compost samples (panel C).

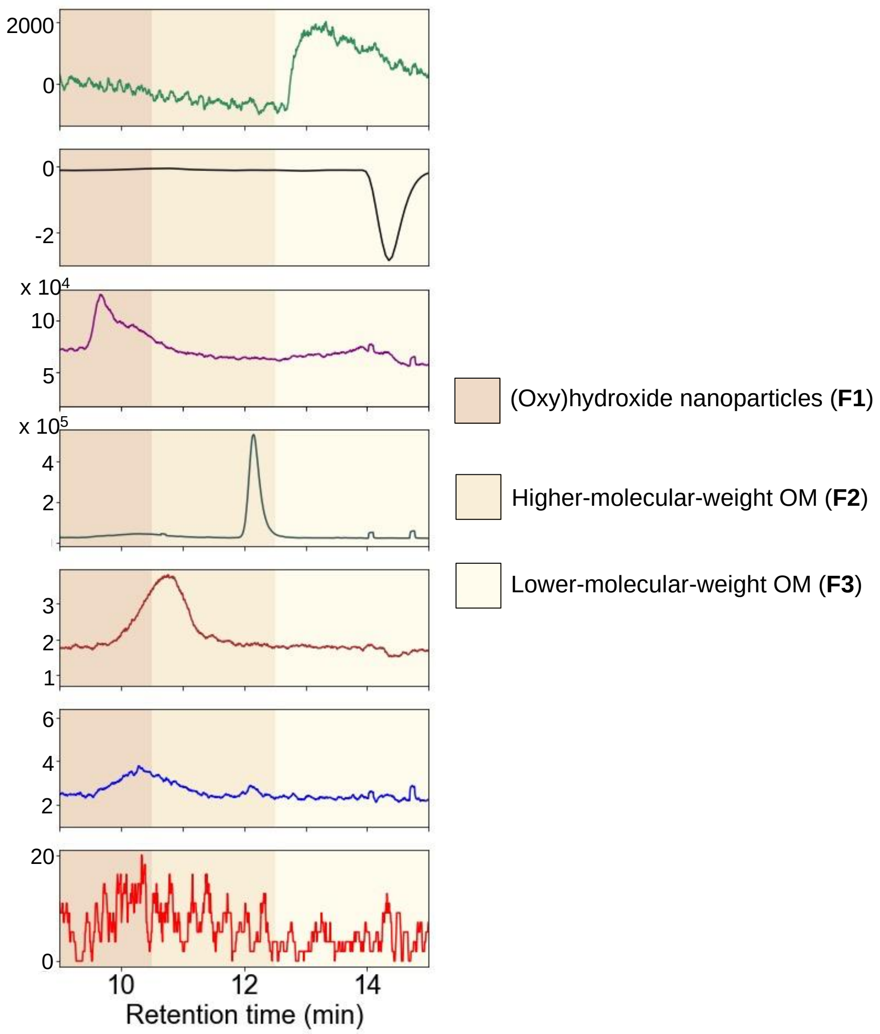

Figure S8. UV and element chromatograms of a water blank measured by SEC-UV-ICP-MS/MS, provided in the following order from top to bottom: C, UV absorbance, P, S, Fe, Zn and Cd. Main S peak is assumed to be evidence of sulfate present in blank.

.

Table S12. Output of the hierarchical cluster analysis performed on WHAM-based and SEC-UV-ICP-MS/MS-based datasets. Input datasets were based on the percentage of water-soluble Zn and Cd in the aqueous inorganic form vs. bound to DOM (derived from WHAM VII) and the percentage of Zn and Cd in SEC fractions F2-3. F1 was not included in cluster analysis calculation due to lack of signal detected in this fraction. Analysis was performed using Ward linkages in SPSS Statistics23 from IBM. Solutions for selection of four, five, and six clusters are presented. The division lines in the table highlight the number of clusters presented in the main text (i.e., 4 clusters). “Lignified crop residues” is abbreviated “LCR”. “Farmyard manure” is abbreviated “FYM”. “Industrial compost” here refers to “compost” in main manuscript.

Sample type	Sample name	Members (6 clusters)	Members (5 clusters)	Members (4 clusters)
Green manure	Berseem clover	1	1	**1**
Green manure	Phacelia	1	1	**1**
Green manure	Green manure mix 1	1	1	**1**
Green manure	Green manure mix 2	1	1	**1**
LCR/Litter	Poultry litter	1	1	**1**
LCR/Litter	Wheat straw	1	1	**1**
Green manure	Yellow mustard	2	2	**2**
LCR/Litter	Barley straw	2	2	**2**
Monogastric FYM	Pig manure (fresh)	2	2	**2**
Industrial compost	Gerber compost 1	2	2	**2**
Industrial compost	Biomassehof compost	3	3	**2**
LCR/Litter	Shredded corn	3	3	**2**
Ruminant FYM	Mixed manure pile	3	3	**2**
Green manure	Subterranean clover	3	3	**2**
Green manure	Black oat	3	3	**2**
Industrial compost	Gerber compost 2	3	3	**2**
Monogastric FYM	Poultry manure	3	3	**2**
Ruminant FYM	Cattle manure	4	4	**3**
Ruminant FYM	Composted cattle manure 1	4	4	**3**
Ruminant FYM	Composted cattle manure 2	4	4	**3**
Ruminant FYM	Composted cattle manure 3	4	4	**3**
Industrial compost	Gerber compost 3	4	4	**3**
Cattle slurry	Cattle slurry 1	5	4	**3**
Cattle slurry	Cattle slurry 2	5	4	**3**
Cattle slurry	Cattle slurry 3	5	4	**3**
Monogastric FYM	Pig slurry	5	4	**3**
Ruminant FYM	Horse manure	5	4	**3**
Monogastric FYM	Pig manure (pile)	6	5	**4**

References

(1) Gerber, L.; Eliasson, M.; Trygg, J.; Moritz, T.; Sundberg, B. Multivariate Curve Resolution Provides a High-Throughput Data Processing Pipeline for Pyrolysis-Gas Chromatography/Mass Spectrometry. *Journal of Analytical and Applied Pyrolysis* 2012, *95*, 95–100. https://doi.org/10.1016/j.jaap.2012.01.011.

(2) Tolu, J.; Gerber, L.; Boily, J.-F.; Bindler, R. High-Throughput Characterization of Sediment Organic Matter by Pyrolysis–Gas Chromatography/Mass Spectrometry and Multivariate Curve Resolution: A Promising Analytical Tool in (Paleo)Limnology. *Analytica Chimica Acta* 2015, *880*, 93–102. https://doi.org/10.1016/j.aca.2015.03.043.

(3) Laborda, F.; Bolea, E.; Górriz, M. P.; Martín-Ruiz, M. P.; Ruiz-Beguería, S.; Castillo, J. R. A Speciation Methodology to Study the Contributions of Humic-like and Fulvic-like Acids to the Mobilization of Metals from Compost Using Size Exclusion Chromatography–Ultraviolet Absorption–Inductively Coupled Plasma Mass Spectrometry and Deconvolution Analysis. *Analytica Chimica Acta* 2008, *606* (1), 1–8. https://doi.org/10.1016/j.aca.2007.10.048.

(4) Grüter, R.; Meister, A.; Schulin, R.; Tandy, S. Green Manure Effects on Zinc and Cadmium Accumulation in Wheat Grains (Triticum Aestivum L.) on High and Low Zinc Soils. *Plant Soil* 2018, *422* (1–2), 437–453. https://doi.org/10.1007/s11104-017-3486-4.

(5) Meng, J.; Wang, L.; Zhong, L.; Liu, X.; Brookes, P. C.; Xu, J.; Chen, H. Contrasting Effects of Composting and Pyrolysis on Bioavailability and Speciation of Cu and Zn in Pig Manure. *Chemosphere* 2017, *180*, 93–99. https://doi.org/10.1016/j.chemosphere.2017.04.009.

(6) Yan, W.; Qu, J.; Qu, Y.; Yue, T.; Zhang, Q.; Yi, W.; Liu, X.; Sun, Y. Effect of Biochar Addition on Mechanism of Heavy Metal Migration and Transformation in Biogas Residue Aerobic Compost. *Fermentation* 2022, *8* (10), 523. https://doi.org/10.3390/fermentation8100523.

(7) Azhar, M.; Zia ur Rehman, M.; Ali, S.; Qayyum, M. F.; Naeem, A.; Ayub, M. A.; Anwar ul Haq, M.; Iqbal, A.; Rizwan, M. Comparative Effectiveness of Different Biochars and Conventional Organic Materials on Growth, Photosynthesis and Cadmium Accumulation in Cereals. *Chemosphere* 2019, *227*, 72–81. https://doi.org/10.1016/j.chemosphere.2019.04.041.

(8) Hölzel, C. S.; Müller, C.; Harms, K. S.; Mikolajewski, S.; Schäfer, S.; Schwaiger, K.; Bauer, J. Heavy Metals in Liquid Pig Manure in Light of Bacterial Antimicrobial Resistance. *Environmental Research* 2012, *113*, 21–27. https://doi.org/10.1016/j.envres.2012.01.002.

(9) Zhang, F.; Li, Y.; Yang, M.; Li, W. Content of Heavy Metals in Animal Feeds and Manures from Farms of Different Scales in Northeast China. *Int. J. Environ. Res. Public Health* 2012, *9* (8), 2658–2668. https://doi.org/10.3390/ijerph9082658.

(10) Provolo, G.; Manuli, G.; Finzi, A.; Lucchini, G.; Riva, E.; Sacchi, G. Effect of Pig and Cattle Slurry Application on Heavy Metal Composition of Maize Grown on Different Soils. *Sustainability* 2018, *10* (8), 2684. https://doi.org/10.3390/su10082684.

(11) Poulsen, P. H. B.; Magid, J.; Luxhøi, J.; De Neergaard, A. Effects of Fertilization with Urban and Agricultural Organic Wastes in a Field Trial – Waste Imprint on Soil Microbial Activity. *Soil Biology and Biochemistry* 2013, *57*, 794–802. https://doi.org/10.1016/j.soilbio.2012.02.031.

(12) Greenway, G. M.; Song, Q. J. Heavy Metal Speciation in the Composting Process. *J. Environ. Monitor.* 2002, *4* (2), 300–305. https://doi.org/10.1039/b110608m.

(13) Tolu, J.; Bouchet, S.; Helfenstein, J.; Hausheer, O.; Chékifi, S.; Frossard, E.; Tamburini, F.; Chadwick, O. A.; Winkel, L. H. E. Understanding Soil Selenium Accumulation and Bioavailability through Size Resolved and Elemental Characterization of Soil Extracts. *Nat Commun* 2022, *13* (1), 6974. https://doi.org/10.1038/s41467-022-34731-6.

(14) Hawkes, J. A.; Sjöberg, P. J. R.; Bergquist, J.; Tranvik, L. J. Complexity of Dissolved Organic Matter in the Molecular Size Dimension: Insights from Coupled Size Exclusion Chromatography Electrospray Ionisation Mass Spectrometry. *Faraday Discuss.* 2019, *218*, 52–71. https://doi.org/10.1039/C8FD00222C.

(15) Persson, L.; Alsberg, T.; Kiss, G.; Odham, G. On-line Size-exclusion Chromatography/Electrospray Ionisation Mass Spectrometry of Aquatic Humic and Fulvic Acids. *Rapid Communications in Mass Spectrometry* 2000, *14* (4), 286–292. https://doi.org/10.1002/(SICI)1097-0231(20000229)14:4<286::AID-RCM879>3.0.CO;2-C
